# Religion and Morality

**DOI:** 10.1037/a0038455

**Published:** 2014-12-22

**Authors:** Ryan McKay, Harvey Whitehouse

**Affiliations:** 1 ARC Centre of Excellence in Cognition and its Disorders, Department of Psychology, Royal Holloway, University of London; 2 Institute of Cognitive and Evolutionary Anthropology, School of Anthropology, University of Oxford

**Keywords:** cognitive science of religion, moral foundations theory, prosocial behavior, cultural evolution

## Abstract

The relationship between religion and morality has long been hotly debated. Does religion make us more moral? Is it necessary for morality? Do moral inclinations emerge independently of religious intuitions? These debates, which nowadays rumble on in scientific journals as well as in public life, have frequently been marred by a series of conceptual confusions and limitations. Many scientific investigations have failed to decompose “religion” and “morality” into theoretically grounded elements; have adopted parochial conceptions of key concepts—in particular, sanitized conceptions of “prosocial” behavior; and have neglected to consider the complex interplay between cognition and culture. We argue that to make progress, the categories “religion” and “morality” must be fractionated into a set of biologically and psychologically cogent traits, revealing the cognitive foundations that shape and constrain relevant cultural variants. We adopt this fractionating strategy, setting out an encompassing evolutionary framework within which to situate and evaluate relevant evidence. Our goals are twofold: to produce a detailed picture of the current state of the field, and to provide a road map for future research on the relationship between religion and morality.

It is simply impossible for people to be moral without religion or God.

—Laura Schlessinger (quoted in Zuckerman, 2008)

Faith can be very very dangerous, and deliberately to implant it into the vulnerable mind of an innocent child is a grievous wrong.

—Richard Dawkins (2006, p. 348)

The question of whether or not morality requires religion is both topical and ancient. In the *Euthyphro*, Socrates famously asked whether goodness is loved by the gods because it is good, or whether goodness is good because it is loved by the gods. Although he favored the former proposal, many others have argued that morality is dictated by—and indeed unthinkable without—God: “If God does not exist, everything is permitted” (Dostoevsky, 1880/1990).^1^ Echoing this refrain, conservatives like to claim that “declining moral standards” are at least partly attributable to the rise of secularism and the decline of organized religion (see Zuckerman, 2008).

The notion that religion is a precondition for morality is widespread and deeply ingrained. More than half of Americans share Laura Schlessinger’s belief that morality is impossible without belief in God (Pew Research Center, 2007), and in many countries this attitude is far more prevalent (see Figure 1). In a series of compelling recent studies, Gervais and colleagues (Gervais, Shariff, & Norenzayan, 2011; see also Gervais, 2011, 2013a, 2014a; Gervais & Norenzayan, 2012b, 2013) have demonstrated strong *implicit* associations of atheists with immorality. Although these associations are stronger in people who themselves believe in God, even atheist participants intuitively view acts such as serial murder, incest, and necrobestiality as more representative of atheists than of other religious, ethnic, or cultural groups (Gervais, 2014a).^2^ Unsurprisingly, atheists explicitly disavow this connection, with some even suggesting that atheists are “the moral backbone of the nation . . . tak[ing] their civic duties seriously precisely because they don’t trust God to save humanity from its follies” (Dennett, 2003). Other nontheists have taken a softer line, arguing that moral inclinations are deeply embedded in our evolved psychology, flourishing quite naturally in the absence of religious indoctrination (Pyysiäinen & Hauser, 2010).[Fig-anchor fig1]

Although there is no shortage of lively polemic, scientific investigations of the connection between religion and morality have so far produced mixed results. The interpretive difficulties are exacerbated by imprecise conceptions both of “religion” and “morality.” It is not clear that these terms are used in the same ways by those between, or even within, seemingly opposing camps. To make progress on this issue, we require a more precise specification of which human virtues are under consideration and which features of religion might be thought to influence their expression. Our aim in what follows will be to sort out some of the conceptual confusions and to provide a clear evolutionary framework within which to situate and evaluate relevant evidence.

We begin by highlighting a set of conceptual limitations hampering contemporary academic discourse on this topic. In our view, many current investigations suffer from (a) a failure to fractionate “religion” and “morality” into theoretically grounded units; (b) ethnocentric conceptions of religion and morality; in particular, (c) sanitized conceptions of prosocial behavior, and (d) a tendency to conceptualize morality or religion as clusters of *either* cognitively *or* culturally evolved features rather than both. To circumvent these problems, we advocate a cross-culturally encompassing approach that fractionates both religion and morality while carefully distinguishing cognition from culture. A thoroughgoing exploration of the religion–morality relationship must seek to establish the evolved cognitive systems that underpin the astonishing diversity of cultural concepts, norms, and behaviors that are labeled (perhaps arbitrarily) “religion” and “morality.” Accordingly, drawing on moral foundations theory (MFT; e.g., Graham et al., 2013), we outline sets of cognitive systems commonly associated with these concepts and consider whether their evolutionary histories might be somehow entwined. We go on to consider the quite separate question of whether the evolution of religions as cultural systems has selectively favored moral values of various kinds. In the process, we provide a comprehensive review of research on the religion–morality relationship.

## Conceptual Lacunae and Confusions in the Religion and Morality Debate

Despite the confident claims of many contemporary commentators, we believe the relationship between religion and morality is poorly understood. In our view, this is because debates about religion and morality are marred by a set of interrelated conceptual lacunae and confusions. Our aim in this section is to enumerate these shortcomings and to highlight some of their serious consequences.

### Astrologizing

History can be written at any magnification. One can write the history of the universe on a single page, or the life cycle of a mayfly in 40 volumes.

—Norman Davies (1997, p. 1)

Just as history can be written at any magnification, the relationship between religion and morality can be explored at any granularity. At the extremes, one can treat “religion” and “morality” as monolithic entities and attempt to characterize their relationship, or one can study the influence of a particular theological doctrine (e.g., predestination) on some highly specific moral outcome (e.g., tithing). The challenge is to adopt a pragmatic and theoretically defensible scale of analysis. One problem with the coarse-grained (monolithic) approach is that religion, like the constellation Orion in the night sky, may not reflect a real natural structure but may instead comprise a more or less arbitrary gathering of disparate features. Researchers in the discipline of cognitive neuropsychiatry view psychiatric syndromes as culturally and historically contingent constellations of symptoms, and argue that the unit of investigation should be the symptom (e.g., delusions) rather than the syndrome (e.g., schizophrenia; Coltheart, Langdon, & McKay, 2011). Likewise, progress in understanding the relationship between religion and morality may require fractionating these hazy concepts into more basic units.

Many authors have attempted to identify the fundamental elements of religion. Saroglou (2011), for instance, has put forward a detailed psychological model of the “Big Four religious dimensions,” providing an illuminating taxonomy of core components of religiosity that integrates numerous previous formulations in the psychology and sociology of religion. In brief, for Saroglou, to be religious entails

1 Believing: Holding a set of beliefs about transcendent entities (e.g., personal gods, impersonal life forces, karmic principles).
2 Bonding: Having self-transcendent, emotional experiences, typically through ritual (whether private or public, frequent or rare), that connect one to others and to a deeper reality.
3 Behaving: Subscribing to certain moral norms, and exerting self-control to behave in accordance with these norms.
4 Belonging: Identifying and affiliating with a certain community or tradition.

Note that any one of these dimensions could pick out phenomena that would not ordinarily be classed as “religious.” For instance, “Father Christmas” is a person who manifestly transcends ordinary physical laws, yet few would describe belief in this supernatural being as “religious” (J. L. Barrett, 2008). Much the same could be said about *ritual*, which is often understood to be a religious trait but is also prominent in nonreligious (e.g., military) settings (and, as Bloom, 2012, notes, even ardent atheists seek out transcendent experiences, whether through drugs or meditative practices). Moreover, Saroglou himself points out that religious affiliation is just one of many ways people can satisfy a need to “belong.”

These considerations point to the arbitrariness of the “religion” designator. Tendencies to postulate bodiless agents such as ghosts and gods and to participate in rituals may seem to warrant some overarching label, but in reality their cognitive causes may be quite unrelated. For example, afterlife beliefs and rituals may be explicitly connected by more or less shared systems of meaning, expressed in discourse at social events like funerals and wakes; and they may form part of larger cultural systems that are transmitted across populations and handed down over generations. But the psychological mechanisms that generate and underpin afterlife beliefs may operate quite independently from those inducing us to perform rituals (Boyer, 2001; Whitehouse, 2004). We should not, therefore, expect the different component features of “religion” each to bear the same connection to morality.

Moreover, according to a prevailing conception in moral psychology, morality—perhaps like religion—comprises a suite of largely independent mechanisms that, although often connected by narratives, doctrines, songs, and other culturally distributed networks of ideas, are the outcomes of quite distinct psychological processes and functions. Thus, both religion and morality can be endlessly assembled and reassembled in culturally and historically contingent ways. Like the constellations of the astrologer’s imagination, these assemblages of psychological and behavioral traits and tendencies may be artificial, contingent, and arbitrary, rather than grounded in any stable underlying regularities (Boyer, 2001; Norenzayan, 2014).

One notable feature of Saroglou’s model of religious dimensions is that it categorizes morality as a key dimension of religion: “Religion not only is particularly concerned with morality as an external correlate but also includes morality as one of its basic dimensions” (Saroglou, 2011, p. 1326). This stipulation implies that any inquiry into the effects of “religion” as a whole *on* “morality” as a whole may be a circular, and therefore futile, enterprise.

### Descriptive Ethnocentrism

If moral psychology is to contribute to the psychology of religion, it will have to describe a moral domain as expansive as that of the Gods.

—Graham and Haidt (2010, p. 143)

When a newspaper headline reads “bishop attacks declining moral standards,” we expect to read yet again about promiscuity, homosexuality, pornography, and so on, and not about the puny amounts we give as overseas aid to poorer nations, or our reckless indifference to the natural environment of our planet.

—Singer (2002, p. 7)

In a recent interview, the Hon. Rev. Fr. Simon Lokodo, Ugandan Minister of Ethics and Integrity, indicated that he viewed the heterosexual rape of young girls as preferable to consensual homosexuality:

Lokodo I say, let them do it but the right way.

Interviewer Oh let them do it the right way? Let them rape children the right way? What are you talking about?

Lokodo No I am saying, at least it is [the] natural way of desiring sex. (O’Brien, 2013)

From a contemporary Western liberal perspective, there is a chilling irony to the fact that Lokodo’s ministerial portfolio involves upholding moral values and principles (see http://www.dei.go.ug). What could be more immoral than the rape of a child, a manifestly harmful act? Is it conceivable that Lokodo’s opposition to homosexuality is morally motivated?

One obstacle to a comprehensive understanding of the relationship between religion and morality is the tendency of researchers to privilege their own cultural perspective on what counts as a “moral concern.” Opposing such ethnocentrism is not the same as advocating cultural or moral relativism: We need take no stand here on whether absolute moral standards exist, or whether it is appropriate for citizens of one society to judge the moral standards of another. Our concern is with *descriptive* rather than prescriptive ethnocentrism. There are those who consider appropriate sexual behavior to be of paramount moral importance, and those, like Peter Singer, who think there are more pressing moral concerns. Whatever our ethical evaluations, however, a cross-cultural enquiry into the relationship between religion and morality must expand the moral domain beyond the typical concerns of individuals in Western, educated, industrialized, rich, and democratic (WEIRD) societies (Henrich, Heine, & Norenzayan, 2010), and must consider the effect of religion on any domain that is accorded at least local moral significance. For our purposes, therefore, a moral behavior is not necessarily a behavior that we advocate, but a behavior that is undertaken on putative moral grounds.

We also view descriptive religious ethnocentrism as problematic. In our view, the great variety of culturally distributed concepts and customs that garner the label “religion” are canalized and constrained by a finite, yet disparate, set of biologically endowed cognitive predispositions (Baumard & Boyer, 2013b; Xygalatas & McKay, 2013). As these predispositions constrain, rather than determine, the types of religious systems that different cultures construct, there is enormous cultural variability in their expression, with some traditions emphasizing conformity of belief (orthodoxy) over conformity of practice (orthopraxy) and vice versa (Laurin & Plaks, 2014; Purzycki & Sosis, 2013).^3^ In short, the religious constellation may look quite different from one cultural perspective than it does from another. This may help to explain why “religion” has proven so notoriously difficult to define in a way that merits scholarly consensus (Asad, 1983; Saler, 2000). To avoid this problem, we should resist the assumption that the core features of “religion” in our own culture (the brightest stars in the constellation from one’s own cultural—or academic—standpoint) are the most important or valid.

### Sanitized Conceptions of Morality and Prosociality

Ingroup generosity and outgroup derogation actually represent two sides of the same coin.

—Shariff, Piazza, and Kramer (2014, p. 439)

A frequent consequence of Western liberal ethnocentrism is a sanitized, “family friendly” conception of morality. If Simon Lokodo’s ministerial portfolio seems ironic, this may be because of a Western liberal tendency to equate morality with “warm, fuzzy” virtues like kindness, gentleness, and nurturance, in short, with “niceness.” Thus, many scholars who write about the relationship between religion and morality frame the key question as “Are religious people nice people?” (Morgan, 1983) or “Does religion make you nice?” (Bloom, 2008; see also Malhotra, 2010). In many situations, however, what seems the “right” course of action may not be particularly “nice” (e.g., is it *nice* to punish criminals?); moreover, in certain cultures (e.g., Nazi Germany), “niceness” may even be cast as a vice rather than a virtue (Koonz, 2003). To identify morality with “niceness” is thus to ignore a plethora of moral concerns, motivations, and behaviors.

To illustrate why such sanitizing is problematic scientifically, we note that the most prominent contemporary hypothesis in the literature on religion and morality is the “religious prosociality” hypothesis. Although many papers on “religious prosociality” appear to equate the notions of morality and “prosociality” (e.g., Norenzayan, 2014; Norenzayan & Shariff, 2008; Preston, Ritter, & Hernandez, 2010), some imply that morality is a subcategory of prosociality (e.g., Galen, 2012), whereas others indicate that prosociality is a subcategory of morality (e.g., Preston, Salomon, & Ritter, 2014). In all of these cases, however, prosociality is used to denote voluntary behaviors that intentionally benefit others at personal cost (e.g., helping, comforting, sharing, donating, volunteering)—in other words, “nice” behaviors (notwithstanding that the motivation to engage in the behaviors may be purely egoistic; Saroglou, 2013). Although this usage reflects both popular parlance and a venerable social scientific tradition (Batson & Powell, 2003), we view it as highly confusing.

The problem is that behavior that benefits certain others (and so is “prosocial” in this standard sense) may be detrimental to the wider social group. And conversely, behavior that benefits the group may be harmful to at least some of its members. For example, torture is a powerful mechanism for enforcing and stabilizing social norms, yet torture is often unambiguously detrimental to the recipient. The irony is that behaviors that are literally “prosocial” insofar as they further the interests of a particular social group (e.g., “prosocial aggression”: Sears, 1961; “altruistic punishment”: Fehr & Gächter, 2002, and Shinada, Yamagishi, & Ohmura, 2004; cf. B. Herrmann, Thöni, & Gächter, 2008) may be “antisocial” in the standard social psychological usage (e.g., by harming the norm violator).

This is not even to consider behavior that extends across group boundaries. Some personally costly acts are intended to benefit the ingroup by harming other groups (Choi & Bowles, 2007, refer to such behavior as “parochial altruism”; see also Bernhard, Fischbacher, & Fehr, 2006; Bowles, 2009; De Dreu et al., 2010). If attendance at religious services predicts support for suicide attacks (Ginges, Hansen, & Norenzayan, 2009), is this evidence for “religious prosociality” or evidence against it? In social psychological terms, it is clearly the latter, but we regard this usage of the term as unhelpfully sanitized. As the saying goes, “One man’s terrorist is another’s freedom fighter” (Seymour, 1975). In an otherwise highly illuminating recent article, social psychologists Jesse Preston and Ryan Ritter referred to cooperation with both ingroup and outgroup members as “prosociality,” while noting that helping outgroup members can give that group a competitive advantage in survival and so indirectly harm the ingroup. Here, behavior that was explicitly acknowledged to harm the ingroup was labeled “prosocial” (Preston & Ritter, 2013). In a different example, Blogowska, Lambert, and Saroglou (2013) found that self-reported religiosity predicted helping of a needy in-group member and also physical aggression toward a member of a moral out-group (a homosexual person). Blogowska et al. described the latter behavior as “clearly and unambiguously” antisocial (p. 525). We argue that this behavior can be reconstrued as (literally) prosocial—after all, if homosexuality is a norm violation from the perspective of a religious group, then behavior that punishes this violation serves to enforce the norm and thus promotes and protects the interests and values of the group.

If the relationship between religion and morality is to be explored within an encompassing evolutionary framework (as we intend), the notion of prosociality should assume a literal rather than sanitized meaning (i.e., “furthering the interests of the relevant social group” rather than “nice”) within an expansive moral domain. As we will describe later, we advocate a strategy of scientific pluralism where morality is concerned. In our view, sanitized prosociality (“caring” or “niceness”) is a core moral domain, but should not be solely identified with “morality.”

### Cognitive Versus Cultural Levels of Explanation

Efforts to fully characterize the relationship between religion and morality are limited by a tendency for researchers to conceptualize morality or religion as bundles of *either* cognitively *or* culturally evolved traits rather than both. For example, Bloom (2012) has attempted to refute the claim that morality requires religion using evidence of (proto)moral behavior in infant humans and in other primates. This argument operationalizes morality at the level of evolved psychological systems, but operationalizes religion as a set of cultural notions. To the extent that “religion” is assumed to refer to some cluster of features that must be culturally learned, this argument may have something to commend it, but at least some of the psychological states that Bloom considers religious (e.g., “spirituality”) are rooted in very early emerging cognitive capacities (J. L. Barrett, 2012). So, in principle, it should be possible to investigate the relationship between at least some aspects (or “building blocks”) of religion and morality in infancy and perhaps also in nonhuman primates.

One way of avoiding this problem is to disambiguate epigenetic, cognitive–developmental, and social–historical processes in the formation of religious and moral traits (Whitehouse, 2013). For example, a capacity to empathize with the pain of others may be genetically canalized in the development of infant neural structures, but environmental cues also shape the organization of neural networks and even the gross morphology of the brain. The interaction of genetic and epigenetic factors in the maturation of empathizing capacities may follow different developmental pathways in different individuals, resulting in quite different outcomes at the level of cognitive and behavioral patterns in adulthood. At a still higher level of complexity, the environment in which brains and cognitive systems develop is itself canalized by social structures comprising culturally distributed rules and algorithms for “proper” or “normal” behavior in given social settings, counterbalanced by population-level decision making on the ground that may deviate from tradition and consequently update its edicts. Processes at all these levels contribute to the nature and targets of empathy in society, influencing people’s willingness to tolerate harming behaviors such as warfare, enslavement, capital punishment, and torture, and calibrating what counts as justice or wanton cruelty. The same principles apply to the development of religious traits. For example, a genetically canalized tendency to process information about mental and mechanical events via quite different neural structures may undergird the cognitive developmental pathways for mind–body dualism (Bloom, 2004), but this tendency is also shaped and constrained by cultural concepts and their histories. When asking, for example, how notions of bodiless agents might impact the development of empathy, we need to specify the level(s) at which the impact is hypothesized to occur and trace its repercussions at all levels on both sides of the religion–morality equation.

## Religion and Morality: A New Approach

In order to circumvent these limitations and avoid these problems, we propose a new approach to the religion–morality debate that not only fractionates both religion and morality but is careful to distinguish the different levels at which explanation is required. This will provide the basis for more precise questions about the relationship between the fractionated components of religion and morality, respectively.

A comprehensive explanation in evolutionary terms of any causal relationships between our fractionated components of the categories “religion” and “morality” would need to attend to four main types of questions, commonly known as Tinbergen’s Four Whys: a causal why, concerning the psychological mechanisms that produce a particular causal relationship between religion and morality; a developmental why, concerning the processes by which the relationship emerges in the growth and maturation of individuals; a functional why, concerning the adaptive value of the relationship in comparison with others; and an historical why, concerning the phylogeny of the relationship, its appearance via a succession of preceding forms (cf. Tinbergen, 1963).^4^ Evolutionary theorists standardly categorize the causal and developmental whys as forms of “proximate” explanation, and the functional and phylogenetic whys as forms of “ultimate” explanation (see Mayr, 1961). In this context, “ultimate” does not mean final or superior, but refers to the evolutionary forces that sustain the psychological or physiological mechanisms in question. Thus, if the pigmentation of butterfly wings in industrial areas becomes darker over successive generations (Haldane, 1927), it is because darker variants have a selective advantage in smoke-stained environments, but that does not dispense with the need to explain the physiological mechanisms by which individual butterfly wings acquire their coloration, darkness, and hue.

Tinbergen’s Four Whys have been illustrated concisely using the structural properties of the human hand:

In answering the question “Why does the human thumb move differently from the other fingers?” the answer might be in terms of the differences in skeletal arrangements and muscle attachments (a causal answer); or in terms of the embryology of the hand, and how the finger rudiments grew out (developmental); or in terms of the utility of an opposable thumb for holding things (functional); or in terms of our descent from monkey-like ancestors which had opposable thumbs (evolutionary). These answers are all correct, but together they provide fuller understanding. (Hinde, 2005, p. 39)

In considering human traits, however, the situation is often complicated by the extent and variability of cultural overlays. In some cases, these are quite literally overlays—for example, in cold environments, human hands may be overlaid by clothing, such as gloves or mittens.

**Figure 2 fig2:**
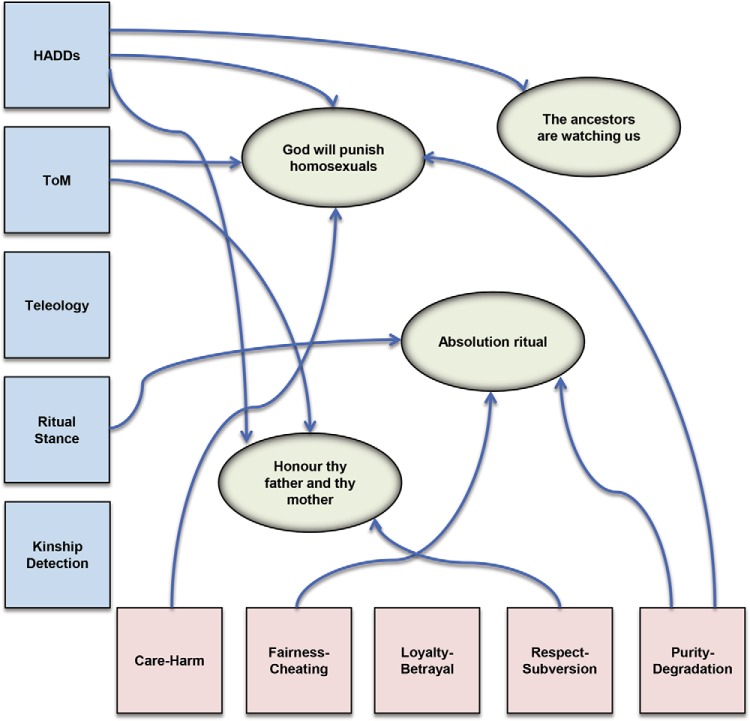
Cultural representations (e.g., propositions, prescriptions, and practices [ovals; green]) are triggered and constrained (arrows; blue) by foundational cognitive systems (“religious foundations” in blue [on the *y* axis] boxes and “moral foundations” in pink [on the *x* axis] boxes). For instance, the proposition that “God will punish homosexuals” may resonate with intuitions of observing, intentional agents, and concerns about harm and purity. The relations depicted here are intended to be illustrative rather than exhaustive. See the online article for the color version of this figure.

Our general theoretical approach melds recent theorizing in disciplines such as moral psychology and the cognitive science of religion. According to this approach, religious and moral cultural representations are triggered and constrained by implicit, intuitive cognitive systems in much the same way that the morphologies of human hands and feet shape and constrain the morphologies of gloves and shoes (see Figure 2). To become culturally widespread, shoes must fit the basic morphology of human feet, while also satisfying other biologically endowed preferences (e.g., preferences for comfort and/or gait; Morris, White, Morrison, & Fisher, 2013). Similarly, successful religious and moral cultural representations—including notions of supernatural agents and realms, ritual practices, and various behavioral prescriptions and proscriptions—must resonate with (“fit”) biologically endowed cognitive structures and preferences (or clash with them in attention-grabbing and memorable ways; see Boyer, 2001). But such structures may, in turn, be subject—given sufficient time scales—to genetic modification under the selection pressures imposed by culturally evolved practices and preferences. A cultural preference for small feet in women may make it more likely that females with such feet are chosen as sexual partners or less likely that they become victims of infanticide (Newson, Richerson, & Boyd, 2007). So just as shoes adapt to the needs of biologically endowed feet, so feet may need to adapt to fit cultural prescriptions. And in the same way, certain universal features of our biologically evolved cognitive architecture and our culturally evolved religious and moral representations may result from complex processes of coevolution. At the risk of mixing metaphors, our minds can be thought of as “fertile ground” for certain cultural representations, “seeds” that “take root in individual human beings . . . and get those human beings to spread them, far and wide” (Dennett, 2006, p. 2). To analyze these various processes correctly, however, it is vital that we disambiguate at which levels selection acts on which traits.[Fig-anchor fig2]

Given this complex interplay between sets of evolved cognitive systems and cultural elements (some of which may be arbitrarily designated “religion” and some arbitrarily designated “morality”), what can it mean to investigate the relationship between religion and morality? In what follows, we begin by fractionating, first, morality and, then, religion into elements that are thought to be recurrent features of human evolved psychology. We then consider whether there is evidence that any of the fractionated elements of religion have a biologically evolved connection to the fractionated elements of morality. We will argue that there is scant evidence for this at present. We then consider the cultural evolution of the religion–morality relationship. Here we argue that cultural evolution has served to connect the fractionated elements of religion and morality in a cascading myriad of ways, and it is at this level primarily that the religion–morality debate might be most fruitfully focused in future.

## Fractionating Morality: Moral Foundations

For the purposes of fractionating morality, we import what we regard as the dominant model in contemporary moral psychology: moral foundations theory (MFT; Graham & Haidt, 2010; Graham et al., 2013; Graham, Haidt, & Nosek, 2009; Haidt, 2012; Haidt & Graham, 2007, 2009; Haidt & Joseph, 2004, 2007). MFT is an avowedly pluralistic theory of morality. Whereas some prominent theorists have favored a “monistic” conception of morality, whereby all moral norms reduce to a single basic moral concern such as “care” or “justice” (e.g., Gray, Young, & Waytz, 2012; Kohlberg, 1971), others (e.g., Berlin, 2013; Gilligan, 1982) have argued there are two or more fundamental, mutually incompatible, and incommensurable moral values. MFT falls within the latter tradition, proposing that the rich array of culturally constructed moral norms and institutions are triggered and constrained by several universal and innate psychological systems—the eponymous moral foundations.

Moral foundations theorists have highlighted five core foundations, giving rise to the following pan-human principles: (a) care–harm: harming others is wrong, whereas treating others with kindness and compassion is right; (b) fairness–cheating: people should reap what they sow and not take more than they deserve; (c) in group loyalty–betrayal: what is good for the community comes above selfish interests; (d) respect for authority–subversion: we should defer to our elders and betters and respect tradition; and (e) purity–degradation: the body is a temple and can be desecrated by immoral actions and contaminants.

Moral foundations theorists claim that each of these principles is written into our distinctively human nature, arising from the normal operation of evolved cognitive mechanisms. On the other hand, the moral foundations are conceived as constraining, rather than determining, the types of moral systems that humans construct. One of the major contributions of the moral foundations approach has been to highlight the cultural and political variability in the expression of these foundations. Some cultures construct their moral norms and institutions on a comparatively small subset of foundations.^5^ For example, whereas the moral orders of most traditional societies are broad, the moral domain in WEIRD cultures (Henrich et al., 2010) is built largely on the first two (“individualizing”) foundations, focusing on the protection of individuals from harm and exploitation (Graham et al., 2013). Meanwhile, a number of studies have found that political liberals value the individualizing principles of care and fairness more than conservatives, whereas conservatives value the “binding” principles of loyalty, authority, and sanctity more than liberals (e.g., Graham et al., 2009; Graham, Nosek, & Haidt, 2012).

Although MFT is not without its critics, we regard it as the most fully developed, integrative, and comprehensive theory of morality currently available. Much criticism to date has focused on MFT’s pluralism (Graham et al., 2013). Some critics (monists) dispute pluralism per se. For example, Gray et al. (2012) have argued that concern about interpersonal harm is the distilled essence of morality, and thus that care/harm is the one true moral foundation. Many moral judgments, however, are difficult to understand “through the lens of intention and suffering” (Gray et al., 2012, p. 103). Consider Simon Lokodo’s judgment that homosexuality is immoral. Many have argued that homosexuality is harmful, for instance, harmful to families or to society more generally (e.g., Bryant, 1977). But Gray et al.’s dyadic model of morality explicitly predicts greater concern for immoral acts that cause direct suffering than those that do not. Few could doubt that the rape of a child causes more “direct suffering” than private consensual sex between same-sex partners. Whereas Gray et al.’s monistic perspective has to shoehorn all moral judgments into the same category, MFT’s pluralism enables concern for rape victims and opposition to homosexuality to be viewed as the expression of different moral foundations—the former the expression of the “care” foundation and the latter founded in “binding” concerns for the welfare of the group and perhaps for bodily purity.

To cite another topical example, the social media service Facebook recently attracted criticism for allowing users to post graphic footage of beheadings, while prohibiting photos of videos containing nudity (including images of breastfeeding in which the baby does not totally obscure the nipple or in which the non-nursing breast is in view; see Clark, 2013). Given the amnesty for posting images of violent murder, it is difficult to see the proscription on breastfeeding images “through the lens of interpersonal harm” (Gray et al., 2012, p. 110). A final example concerns moral judgments of suicide, the self-directed nature of which poses an apparent problem for Gray et al.’s dyadic model. One might argue that people who commit suicide harm others (e.g., loved ones) as well as themselves, and that the harm to others is the source of disapprobation when suicide is concerned. However, a recent study by Rottman, Kelemen, and Young (2014) casts doubt on this explanation. Participants read a series of fictitious (but ostensibly real) obituaries describing suicide or homicide victims, and made a series of ratings (including rating the moral wrongness of each death). Whereas perceived harm was the only variable predicting moral judgments of homicide, feelings of disgust and purity concerns—but *not* harm ratings—predicted moral condemnations of suicide. Thus, contrary to participants’ explicit beliefs about their own moral judgments, suicide was deemed immoral to the extent that it was considered impure.

Other critics of MFT’s pluralism have not questioned the idea of pluralism per se, but have objected to MFT’s particular brand of pluralism. However, proponents of MFT do not claim that their list of five foundations is exhaustive, but have actively sought out arguments and evidence for others (e.g., research is currently underway to evaluate the additional candidates of liberty–oppression, efficiency–waste, and ownership–theft; Graham et al., 2013; Iyer, Koleva, Graham, Ditto, & Haidt, 2012). Moral foundations theorists have put forward their own celestial analogy to describe the process of identifying foundations:

There are millions of objects orbiting the sun, but astronomers do not call them all planets. There are six (including the Earth) that are so visible that they were recorded in multiple ancient civilizations, and then there are a bunch of objects further out that were discovered with telescopes. Astronomers disagreed for a while as to whether Pluto and some more distant icy bodies should be considered planets. Similarly, we are content to say that there are many aspects of human nature that contribute to and constrain moral judgment, and our task is to identify the most important ones. (Graham et al., 2013, pp. 104–105)

Using the fairness foundation for illustration, Graham et al. (2013) provide five criteria that any “aspect of human nature” must satisfy to qualify as a moral foundation. First, the relevant moral concern must feature regularly in third-party normative judgments, wherein people express condemnation for actions that have no direct consequences for them. Fairness certainly satisfies this requirement—as Graham and colleagues note, gossip about group members who violate fairness norms (e.g., who cheat, free ride, or neglect to reciprocate) is ubiquitous in human groups, with some authors even suggesting that gossip between third parties evolved as a mechanism for detecting and dissuading cheating and free riding (e.g., see Ingram, Piazza, & Bering, 2009). Second, violations of the moral principle in question must elicit rapid, automatic, affectively valenced evaluations. LoBue, Nishida, Chiong, DeLoache, and Haidt (2011) found that children as young as 3 years old reacted rapidly and negatively to unequal distributions of stickers, particularly personally disadvantageous distributions.

For Graham et al. (2013), these two criteria establish the “moral” quality of the foundations. Their last three criteria relate to foundationhood per se. First, foundational moral concerns should be culturally widespread. In terms of fairness, a preference for interactions based on proportionality is certainly widespread (Baumard & Boyer, 2013a; Gurven, 2004), and people from a diversity of cultures appear more interested in relative than absolute benefits (Brosnan & de Waal, 2005; Henrich et al., 2005). According to Graham et al., a society has yet to be identified in which reciprocity is not a prominent moral concern. Second, there should be indicators of innate preparedness for foundational concerns. Evidence that capuchin monkeys will sometimes forgo a food reward delivered by an experimenter who has previously paid another monkey a more attractive reward for equal effort (Brosnan & de Waal, 2003) suggests that fairness concerns are found in at least some nonhuman primates. Moreover, developmental studies show that young infants are sensitive to inequity. For example, Sloane, Baillargeon, and Premack (2012) found that 21-month-old children expected an experimenter to reward each of two individuals when both had worked at an assigned task, but not when one of the individuals had done all the work. Baumard, Mascaro, and Chevallier (2012) found that 3- and 4-year-old children were able to take merit into account by distributing tokens according to individual contributions.

Finally, an evolutionary model should clearly specify the adaptive advantage conferred by the candidate foundation upon *individuals* who bore it in the ancestral past (as Graham et al., 2013, note, a good evolutionary theory will not invoke biological *group* selection without adducing a great deal of additional support). Fairness meets this criterion nicely. For example, Baumard, André, and Sperber (2013) have compellingly argued that fairness preferences are adapted to an environment in which individuals competed to be selected and recruited for mutually advantageous cooperative interactions (see also Trivers, 1971).

## Fractionating Religion: Religious Foundations?

Just as it is possible to decompose the category “morality” into a set of theoretically grounded elements, “religion” can be fractionated into distinct components with stable cognitive underpinnings. Research in the “cognitive science of religion” has not sought to demonstrate the universality of any particular religious representations, such as various notions of ancestors, punitive deities, creator beings, or sacrifices, blessings, and rites of passage. Rather, the aim has been to show that the great variety of culturally distributed dogmas and practices that have been collectively labelled “religion” are shaped and constrained by a finite but disparate set of evolved cognitive predispositions—what we might call “religious foundations.” These foundations comprise a set of evolved domain-specific systems, together with the intuitions and predispositions that those systems instill (see Baumard & Boyer, 2013b; also see Figure 2). Barring pathology—itself a valuable source of insight into natural cognition (Coltheart, 1984; Ellis & Young, 1988)—such tendencies emerge in all human beings without the need for deliberate instruction or training, even if their expression in development may be “tuned” by cultural environments (McCauley, 2011).

Although Saroglou (2011) provides a valuable synthesis of previous taxonomies of core religious dimensions, in our view, the dimensions he settles on (Believing, Bonding, Behaving, Belonging) do not correspond well to evolved cognitive systems, so are not good candidates for religious foundations. For example, Saroglou’s Believing dimension encompasses belief in “divine beings” and belief in “impersonal forces or principles” (p. 1323). There are at least two important and potentially dissociable supernatural concepts here: the notion of supernatural *agency*, on the one hand (e.g., gods, spirits, angels, “ancestors”), and the notion that our actions in this life have *proportionate* (Baumard & Boyer, 2013a), supernaturally mediated consequences, on the other. These consequences may be mediated by supernatural agents, as when gods bestow rewards or dispense punishments in this life or the next; but they may also reflect the impersonal unfolding of a cosmic principle (e.g., *Saṃm.sāra*). Moreover, supernatural agents are not necessarily in the business of attending to our behaviors and implementing relevant consequences—as we shall review, gods vary in their concerns with human affairs in general and with moral issues more specifically. In view of these various considerations, one could posit not one but two distinct dimensions of supernatural belief here: (a) supernatural agency, and (b) supernatural justice. Rather than take this route, our preference is to specify a small subset of evolved cognitive systems that, jointly or in isolation, would account for why these dimensions are cross-culturally and historically recurrent.

Here we discuss five strong candidates for religious foundationhood: (a) a system specialized for the detection of *agents*; (b) a system devoted to representing, inferring, and predicting the mental states of *intentional* agents; (c) a system geared toward producing teleofunctional explanations of objects and events; (d) a system specialized for affiliating with groups through the imitation of causally opaque action sequences; and (e) a system specialized for the detection of genetic kinship. Like proponents of MFT, we do not claim that this list is exhaustive, and future research may suggest alternative, or additional, candidates (when relevant, we discuss current alternate views). Our commitment, born of doubt that there is any “distilled essence” of religion (Gray et al., 2012), is primarily to a pluralistic approach. Nevertheless, based on an extensive review of the cognitive science of religion literature, the following represent the most plausible candidates for universal religious foundations, on current evidence.

### Hyperactive Agency Detection

According to error management theory (Haselton, 2003; Haselton & Buss, 2000; Haselton & Nettle, 2006; D. D. P. Johnson, Blumstein, Fowler, & Haselton, 2013; McKay & Efferson, 2010), in any domain characterized by a recurrent asymmetry in the fitness costs of relevant errors, natural selection should favor the evolution of cognitive systems that minimize the more costly error(s). This logic has been used to undergird an influential claim in the cognitive science of religion. Guthrie (1993) has argued that for humans in the ancestral past, mistaking an *agent* (e.g., an approaching predator) for an inanimate object (e.g., a tree rustling in the wind) was more costly than the converse error. Humans should therefore be equipped by natural selection with biased agency-detection mechanisms—what J. L. Barrett (2000, 2004, 2012) has termed “Hyperactive [or hypersensitive] agent-detection devices” (HADDs).

HADDs are often described as perceptual mechanisms, devices biased toward the perception of agents in ambiguous stimulus configurations. A by-product of their functioning would be a tendency toward false positives (e.g., perceiving representations of human or animal figures in arbitrary collections of stars, or “faces in the clouds”; Guthrie, 1993). A broader conception of HADDs includes attributions of nonrandom structure (Bloom, 2007)—such as naturally occurring patterns and events with no clear physical cause—to the *activity* of agents. In other words, HADDs are a suite of hypothetical devices specialized for perceiving either agents or their effects. A corollary of these “proper functions” (Millikan, 2005) would be the postulation of unseen, or fleetingly visible, *supernatural* agents. Such notions, once posited, would be attention grabbing, memorable, and thus highly transmissible because of their resonance with intuitive cognitive structures such as HADDs (J. L. Barrett, 2000; J. L. Barrett & Lanman, 2008). Indeed, just as the cultural success of high-heeled shoes may owe to the fact that they function as supernormal stimuli (insofar as they exaggerate sex specific aspects of female gait; Morris et al., 2013), notions of supernatural agency may represent supernormal stimuli for evolved agency-detection mechanisms.

At present, the evidence for a connection between supernatural concepts and beliefs and agency cognition is mixed. On the one hand, Norenzayan and colleagues (Norenzayan, Hansen, & Cady, 2008; Willard & Norenzayan, 2013) have found that tendencies to anthropomorphize (e.g., to rate natural scenes using agentic concepts) predict paranormal beliefs (i.e., Psi, precognition) but not belief in God (at least not for Christian participants, who may view anthropomorphism as akin to idolatry and may therefore suppress it). Similarly, Van Elk (2013) found that whereas paranormal beliefs were strongly related to a tendency to erroneously identify walking human figures in point-light displays (see also Krummenacher, Mohr, Haker, & Brugger, 2010), traditional religious beliefs were not. However, in a follow-up priming study, van Elk, Rutjens, van der Pligt, and van Harreveld (in press) found that participants’ religiosity moderated the effect of supernatural priming on agency detection, such that religious participants perceived more agents and responded faster to face stimuli following supernatural primes than nonreligious participants. Meanwhile Riekki, Lindeman, Aleneff, Halme, and Nuortimo (2013) found that religious believers showed more of a bias than nonbelievers to indicate that photographs of inanimate scenes (e.g., furniture, buildings, natural landscapes) contained face-like images. In all of these studies, agency detection was a measured variable. As far as we are aware, to date, no published study has investigated whether *manipulating* cues of agency (e.g., watching eyes; see The Cross-Cultural Prevalence of Supernatural Punishment Concepts) can increase religious belief. Given the hypothesized causal route (whereby agency detection biases predispose humans to acquire beliefs in religious concepts), this may be a fruitful avenue for future research.

### Theory of Mind (ToM)

Notions of supernatural beings as psychological entities with beliefs, preferences, and intentions—*intentional* agents—are also likely to be compelling for humans in light of their expertise in representing, inferring, and predicting the mental states of others (ToM; Baron-Cohen, 1995; Mitchell, 2009; Premack & Woodruff, 1978). Recent studies demonstrate a robust relationship between such “mentalizing” capacities and religious cognition (see Gervais, 2013b). For example, functional MRI experiments with religious participants have shown that religious belief (Kapogiannis et al., 2009) and improvised prayer (Schjoedt, Stødkilde-Jørgensen, Geertz, & Roepstorff, 2009) engage neural networks subserving ToM capacities. Moreover, supernatural believers rate the random movements of animated geometric objects as more intentional than skeptics do, and evince stronger activation of ToM-related networks while viewing such animations (Riekki, Lindeman, & Raij, 2014). Finally, Norenzayan, Gervais, and Trzesniewski (2012) found that autistic participants expressed less belief in God than did matched neurotypical controls. In follow-up studies using nonclinical samples, these authors found that higher autism scores predicted lower belief in God, a relationship mediated by mentalizing abilities.

ToM is also thought to play an important role in afterlife beliefs. It has been suggested, for example, that people spontaneously infer that dead relatives and friends are still present, even in the absence of cultural inputs to support such ideas. Bering and colleagues conducted experiments with children (Bering & Bjorklund, 2004; Bering, Blasi, & Bjorklund, 2005) and adults (Bering, 2002) in which participants were presented with scenarios in which specified agents (puppets in the case of the child studies) experienced various sensations, emotions, and thoughts prior to death (e.g., before being gobbled up by a crocodile-shaped puppet). Participants of all ages tended to make “discontinuity judgments” with respect to sensorimotor and perceptual capacities—for example, inferring that a dead agent would immediately lose the ability to walk, taste, smell, and feel hungry. At the same time, however, participants tended to reason that higher level cognitive functions, such as memories, emotions, and beliefs, would continue to function normally, such responses being coded as “continuity judgments” (E. Cohen & Barrett, 2008). Interestingly, this pattern was stronger in younger children, such that continuity judgments across all faculties gradually diminished with age; however, this pattern has not been replicated in some other studies (Astuti & Harris, 2008; Harris & Gimenez, 2005).

Bering’s (2002, 2006) explanation for these psychological findings hinges, in part, on what he calls the “simulation constraint hypothesis” (see Hodge, 2011, for a review). The idea is that although we can simulate the loss of perceptual capacities like sight and hearing simply by covering the relevant organs (the eyes and the ears), we cannot simulate the absence of thoughts, desires, memories, and so on. The proposal is akin to positing a “hyperactive” ToM, which makes it easier to represent minds as persisting, irrespective of what happens to the body (for related ideas, see Bloom, 2004, 2007). Even people who hold explicitly extinctivist beliefs (e.g., who are adamant, when questioned, that personal consciousness is terminated at death) make a striking number of continuity responses with respect to emotional, desire, and epistemic states (Bering, 2002, 2006). The root of this, Bering argues, is that humans have dedicated cognitive machinery for reasoning about mental states, which, unlike our capacities for reasoning about the mechanical and biological properties of bodies, cannot conceptualize total system failure.

If Bering (2002, 2006) is right that humans are incapable of simulating the absence of higher level cognitive functions, and if this putative incapacity is what underlies “continuity judgments,” then one would expect to observe a similar pattern in other scenarios involving a complete lack of sentience or experience. For example, participants should be unable to fully appreciate that people lack conscious experiences when under general anesthesia, or that inanimate objects such as carpets and kitchen utensils lack such experiences. Although we think this is implausible, it is an empirical question whether continuity judgments can be elicited in such scenarios. We note in this connection that recent research on *pre*life beliefs in Ecuadorian children indicates that, until about 9 to 10 years of age, they ascribe several biological and psychological capacities to their prelife selves; moreover, older children, who ascribe fewer capacities to themselves overall, are still more likely to ascribe certain mental states—in particular, emotional and desire states—to their prelife selves than other mental states (e.g., perceptual, epistemic states; Emmons & Kelemen, 2014).

### Teleofunctional Explanations

Another foundational cognitive predisposition where religion is concerned may be a tendency to favor teleofunctional reasoning. Research by Kelemen and colleagues (e.g., Kelemen, 1999a, 1999b, 1999c, 2004) suggests that children display a broad inclination to view objects and behaviors of all kinds—including features of the natural world—as existing for a purpose. For instance, when confronted with multiple accounts of why rocks are “pointy,” children tend to reject explanations that appeal to the effects of long-term erosion by wind and rain, and instead prefer functional accounts such as “rocks are pointy to stop elephants sitting on them.”

Although it may be tempting to think that this teleological bias is attributable simply to acquisition of a creationist worldview (e.g., regular retellings of the Genesis story), several lines of evidence suggest otherwise. For instance, Evans (2001) has found that, irrespective of their community of origin (whether Christian fundamentalist or nonfundamentalist), young children prefer “creationist” explanations of natural phenomena; only later in development do the children of nonfundamentalists diverge from the position that natural phenomena result from nonhuman design. Research conducted with nonschooled Romani adults, who are unfamiliar with scientific accounts of evolutionary origins, arguably demonstrates the persistence of teleological intuitions into adulthood (Casler & Kelemen, 2008). Moreover, elderly patients suffering from Alzheimer’s disease, a condition that erodes semantic memory (including scientific schemas), are more likely to accept and prefer unwarranted teleological explanations than healthy participants (Lombrozo, Kelemen, & Zaitchik, 2007). Finally, university students (Kelemen & Rosset, 2009), and even actively publishing physical scientists (Kelemen, Rottman, & Seston, 2013), demonstrate increased acceptance of teleological explanations of natural phenomena when their information-processing resources are limited. These results suggest that an underlying tendency to construe the world in functional terms is present throughout life (Kelemen & Rosset, 2009). If so, this tendency may render notions of intelligent supernatural designers, who have created the world and everything in it for a purpose, especially compelling (Kelemen, 2004).

Ojalehto, Waxman, and Medin (2013) present an intriguing “relational–deictic” interpretation of this putative teleological bias. According to these authors, although many teleological explanations that children favor may seem “unwarranted” (Kelemen & Rosset, 2009; Kelemen et al., 2013) from a Western, scientific perspective, this is a culturally infused stance. Thus, just as our tendency to speak of the sun as “rising” reflects our particular geocentric perspective on the relation between the earth and the sun, and does not (anymore) represent our abstract beliefs (Purzycki, 2013), an utterance such as “rainclouds are for giving animals water” may reflect an appreciation of the perspectival relations among living things and their environments rather than a deep-seated intuition about context-independent purpose in nature. To the extent that this relational-deictic stance represents a cognitive default, however, it may still serve as a strong foundation for religious cultural notions. In particular, although we agree with Ojalehto et al. (2013, p. 169) that “teleological statements do not necessarily signify a commitment to an intentional creator,” we think it plausible that tendencies to view the world in functional terms—whether the functions in question are intrinsic to entities or relationships—may make notions of purposeful creator beings especially resonant. Recent evidence that acceptance of teleological explanations is related to belief in God, as well as to belief in Nature as a powerful “being” (Kelemen et al., 2013; see also Willard & Norenzayan, 2013), is consistent with this suggestion.

### The “Ritual Stance”

Humans often imitate each other without knowing why—that is, with little or no understanding of how the actions contribute to goals. *Causal opacity* of this kind is a hallmark feature of ritualized behavior. In rituals, the relationship between actions and stated goals (if indeed they are stated at all) cannot, *even in principle*, be specified in physical–causal terms (P. A. Herrmann, Legare, Harris, & Whitehouse, 2013; Whitehouse, 2011). Social anthropologists have often observed that ritual participants are powerless to explain why they carry out their distinctive procedures and ceremonies, appealing only to tradition or the ancestors. But of considerable interest, too, is the fact that nobody has any difficulty understanding the anthropologist’s question, when she asks what the rituals mean. People know that ritualized actions can be invested with functions and symbolic properties even though they may struggle on occasion to identify what those may be, often pointing the hapless researcher in the direction of somebody older or wiser (Humphrey & Laidlaw, 1994; Staal, 1989).

Imitation of causally opaque behavior is a distinctively human trait. None of the other great apes shows a marked interest in devising highly stylized procedures and bodily adornments and using these to demarcate and affiliate with cultural groups. Although chimpanzees and other primates do engage in social learning, they attend preferentially to technically useful skills that transparently contribute to proximal end goals (Call, Carpenter, & Tomasello, 2005). Because rituals lack overt usefulness, most animals would not see any value in copying them. Yet by meticulously conforming to arbitrary social conventions, human groups bind themselves together into cooperative units facilitating cooperation on a scale that is very rare in nature.

From an evolutionary perspective, deriving the benefits of group living requires a means of identifying ingroup members (the ones you should cooperate with) and out-groups (people you should avoid or compete with). One solution is to have a distinctive set of group conventions or rituals (of course, there are other means too, e.g., humans use language to communicate about group identity). When a set of rituals is performed frequently enough, it becomes easy to identify unauthorized innovations, and so the group’s beliefs and practices can be standardized across substantial populations (Whitehouse, 2004).

One of the many clues that ritualistic behavior is written into our species’ evolved biological makeup is the fact that it emerges early in development (Nielsen, 2006). Even infants show considerable interest in causally opaque behavior and will try to copy it (Gergely, Bekkering, & Király, 2002). Indeed, the willingness to copy arbitrary conventions is essential for acquiring language requiring us to accept that arbitrary utterances refer to stable features of the world around us, not because there is a causal relationship between the sound and the thing it refers to, but simply because that is the accepted convention. The human tendency to copy causally opaque behavior is sometimes called “overimitation” (Whiten, McGuigan, Marshall-Pescini, & Hopper, 2009). Psychologists have known for some time that if you show children an unnecessarily complicated way of retrieving an object from a box, they will copy not only the causally necessary behavior but also the useless frills (Lyons, Young, & Keil, 2007). One possibility is that overimitation evolved to help children acquire complex technical skills in the absence of a fuller understanding of their underlying causal structure (Schulz, Hooppell, & Jenkins, 2008). Another possibility is that overimitation is designed to help children learn arbitrary group conventions or “rituals.” Such behavior may be motivated by a desire to belong, rather than to learn anything technically useful (P. A. Herrmann et al., 2013; Kenward, Karlsson, & Persson, 2011). This view is supported by recent research showing that priming ostracism threat increases the propensity to imitate causally opaque action sequences (Watson-Jones, Legare, Whitehouse, & Clegg, 2014).

### Kinship Detection

Inclusive fitness theory predicts that organisms will behave in ways that preferentially benefit kin, with more benefits conferred as the degree of genetic relatedness between the actor and the recipient increases (Hamilton, 1964). Mechanisms for recognizing and calibrating kinship are critical for such behaviors to evolve and can be classified as one of two broad types: those that exploit direct, phenotypic cues (e.g., visual similarity to self), and those that exploit indirect, contextual cues (e.g., coresidence early in life; DeBruine et al., 2011; Penn & Frommen, 2010). According to Lieberman, Tooby, and Cosmides (2007), cues indicative of kinship are taken as input by two separate motivational systems. The first regulates altruistic behaviors toward kin (Krupp, Debruine, & Barclay, 2008), whereas the second regulates sexual attraction and aversion, thereby avoiding the deleterious consequences associated with close inbreeding (Bittles & Neel, 1994).

As Pinker (2012) points out, kin recognition in humans depends on cues (in particular, linguistic cues) that others can manipulate:

Thus people are also altruistic toward their adoptive relatives, and toward a variety of fictive kin such as brothers in arms, fraternities and sororities, occupational and religious brotherhoods, crime families, fatherlands, and mother countries. These faux families may be created by metaphors, simulacra of family experiences, myths of common descent or common flesh, and other illusions of kinship.

Cultural manipulations of kinship detection machinery may be rife in ritualistic behavior. As Saroglou (2011) notes, religious rituals serve to *bond* ritual participants together. Such rituals may accomplish this, in part, by incorporating a range of kinship cues. First, many religious rituals involve artificial phenotypic cues of kinship—similar costumes, headdress, face paint, and so forth. Second, *social synchrony* is a key feature of many religious rituals, and has long been hypothesized to promote group cohesion (e.g., Durkheim, 1915/1965; Turner, 1969/1995). Recent experimental studies confirm that synchronic movement increases cooperation among participants. For example, Wiltermuth and Heath (2009) found that participants who engaged in synchronic behaviors (e.g., walking in step, synchronous singing and moving) contributed more to the public good in subsequent group economic measures than control participants. Fischer, Callander, Reddish, and Bulbulia (2013) investigated nine naturally occurring rituals and found that those which incorporated synchronous body movements increased perceptions of oneness with their group (see also Hove & Risen, 2009; Reddish, Fischer, & Bulbulia, 2013; Valdesolo & DeSteno, 2011; Valdesolo, Ouyang, & DeSteno, 2010). Interpersonal multisensory-stimulation experiments have demonstrated that synchronous stimulation causes participants to perceive others as both more physically and psychologically similar to themselves (Paladino, Mazzurega, Pavani, & Schubert, 2010; Tajadura-Jiménez, Grehl, & Tsakiris, 2012; Tsakiris, 2008).

Third, the *arousal* that many rituals generate may function as a contextual cue to kinship. In particular, coparticipants in intense, *dysphorically* arousing rituals may gain a quantity of “shared experience” normally possible to accumulate only through a large number—perhaps a lifetime—of shared interactions. As a result, such experiences may activate context-based kinship detection mechanisms, contributing to group cohesion (Whitehouse & Lanman, in press). Xygalatas et al. (2013) studied two Hindu rituals in Mauritius—a low-ordeal ritual involving singing and collective prayer, and a high-ordeal ritual involving body piercing, carrying and dragging heavy structures, and climbing a mountain to reach a temple. Following the ritual, participants were paid around two days’ salary for participating in the study and had the opportunity to anonymously donate any part of this money to the temple. High-ordeal participants donated significantly more than low-ordeal participants, and higher levels of self-reported pain were associated with greater donations.

## The Religion–Morality Relationship in Biological Evolution

A key feature of our approach is to consider whether the fractionated components of morality and religion have overlapping evolutionary histories. As noted earlier, just as there are genetically endowed physical structures (e.g., limbs and other bodily appendages) and cultural artifacts (e.g., gloves and hats) that are shaped by (and in turn potentially shape) these structures, so there are genetically endowed cognitive structures (innately specified cognitive mechanisms and intuitions) and cultural concepts (e.g., supernatural concepts, stories, and dogmas) that are shaped by (and potentially shape) these structures. Some of these structures and concepts are (perhaps arbitrarily) designated “religion,” and some as “morality.” Our strategy is, first, to identify some of the key elements of our genetically inherited psychology, and to consider whether there is evidence that any of the elements typically designated as “religion” have a biologically evolved connection to any of the elements typically designated as “morality.” We now have before us two sets of domain-specific evolved psychological systems—a set of putative moral foundations and a set of candidate religious foundations. Our fractionating strategy produces a preliminary matrix of at least 25 basic questions at the level of biological evolution (e.g., “Is there a biologically evolved connection between HADDs and the care/harm foundation?”; “Is there a biologically evolved connection between kin detection mechanisms and the authority/subversion foundation?”).

In our view, the most plausible cases of biologically evolved connections between the religious and moral foundations involve agency-detection mechanisms and ToM. As we have seen, Guthrie’s (1993) proposal is that biased agency-perception mechanisms (assuming they exist) are an adaptation for avoiding predators. If the functioning of such mechanisms led to conclusions about the presence of invisible, *supernatural* agents, this was (at least initially) merely a by-product—a biological spandrel (Gould & Lewontin, 1979). Likewise, if the limitations of our evolved capacities to simulate mental states, *or the absence of such states*, triggered intuitions about the continued (invisible) presence of dead individuals, this would have been incidental. However, D. D. P. Johnson, Bering, and colleagues (e.g., Bering & Johnson, 2005; D. D. P. Johnson, 2009; D. D. P. Johnson & Bering, 2006; D. D. P. Johnson & Krüger, 2004) have suggested that such incidental deliverances may have been exapted for an important function at a later evolutionary stage (an exaptation is a feature whose benefits to the organism that possesses it are unrelated to the reasons for its origination—originally, the feature may have served a different purpose [or no purpose], but later it became coopted for a new purpose; Barve & Wagner, 2013; Gould & Vrba, 1982).

The supposition of moral-foundations theorists is that the various foundations evolved to solve a range of adaptive problems (e.g., as noted earlier, the fairness/cheating foundation is thought to have evolved to procure the benefits of two-way partnerships; Baumard et al., 2013; Graham et al., 2013). The evolution of these various mechanisms would have occasioned a novel set of selection pressures—in particular, the costs associated with being caught violating foundational moral principles. According to D. D. P. Johnson, Bering, and colleagues, the evolution of linguistic and mentalizing capacities would have ramped up these costs, as moral transgressions could be reported to absent third parties, exacerbating reputational damage for the transgressor. The conjunction of these various mechanisms, therefore, may have increased the premium on mechanisms that inhibit moral transgressions. Intuitions about punitive supernatural observers—a short excursion through Design Space (Dennett, 1995) for mechanisms that are already generating ideas about invisible supernatural agents as a matter of course—would fit the bill here: “What better way [to avoid the fitness costs associated with the real-world detection of moral transgressions] than to equip the human mind with a sense that their every move—even thought—is being observed, judged, and potentially punished?” (D. D. P. Johnson, 2009, p. 178).

The notion that humans have a genetically endowed propensity to postulate moralizing, punitive supernatural observers is both compelling and controversial. If intuitions about punitive supernatural observers are a biological mechanism for inhibiting moral transgressions, we should expect activation of these intuitions to have the relevant inhibitory effect. In the next section, we review the evidence for this hypothesis.

### Supernatural Agent Intuitions and Morality

Surveys indicate that people who score higher on indices of religiosity (e.g., frequency of prayer and religious service attendance) reliably report more helping behaviors, such as charitable donations (Brooks, 2006; Putnam & Campbell, 2010). As Norenzayan and Shariff (2008) have persuasively argued, however, this “charity gap” could occur because of an important confound: It may be that religious individuals are simply more motivated to maintain a moral reputation than nonreligious individuals (see also Batson, Schoenrade, & Ventis, 1993; Sablosky, 2014). This would render religious individuals more susceptible to social desirability concerns, to which self-report measures of socially desirable behaviors are notoriously vulnerable (Paulhus, 1991). Indeed, studies have found a consistent empirical link between religion and socially desirable responding (Eriksson & Funcke, 2014; Sedikides & Gebauer, 2010), which raises the prospect that results linking religion with moral behavior largely reflect concerns to present a positive image to the researcher (Galen, 2012; Saroglou, 2012, 2013). Some studies have found that a link between self-reported religiosity and self-reported altruism remains even when social desirability concerns are measured and controlled for (e.g., Saroglou, Pichon, Trompette, Verschueren, & Dernelle, 2005). However, to the extent that the relationship between religiosity and self-enhancement stems from self-stereotyping rather than from concerns with projecting a positive image (Eriksson & Funcke, 2014), attempts to control for socially desirable responding may not eliminate all relevant sources of response bias in self-report measures. Accordingly, experiments with behavioral measures should be consulted wherever possible (Norenzayan & Shariff, 2008).

A growing body of studies have utilized experimental and naturalistic priming paradigms in a bid to uncover causal—rather than merely correlational—relationships between concepts of supernatural agency and morally relevant behaviors (see Norenzayan & Shariff, 2008; Shariff & Norenzayan, 2007).^6^ To date, such studies have found evidence that, compared with control participants, those primed with supernatural concepts are more cooperative in experimental economic measures, such as dictator games (Ahmed & Salas, 2011; Shariff & Norenzayan, 2007; cf. Benjamin, Choi, & Fisher, 2010), public goods games (Ahmed & Hammarstedt, 2011; Benjamin et al., 2010), common-pool resource games (Xygalatas, 2013), and prisoner’s dilemma games (Ahmed & Salas, 2011).^7^ Moreover, primed participants evince greater intention to help others (Malhotra, 2010; Pichon, Boccato, & Saroglou, 2007; Pichon & Saroglou, 2009), less willingness to cheat (Aveyard, 2014; Bering, McLeod, & Shackelford, 2005; Carpenter & Marshall, 2009; Mazar, Amir, & Ariely, 2008; Randolph-Seng & Nielsen, 2007), and greater self-control (Friese & Wänke, 2014; Laurin, Kay, & Fitzsimons, 2012; Rounding, Lee, Jacobson, & Li, 2012; Toburen & Meier, 2010; cf. Harrison & McKay, 2013).^8^

One limitation of some of these behavioral studies, from a pluralistic moral perspective, is that competing moral motivations are sometimes conflated. For example, given the effect of religious priming on dictator game allocations, one might conclude that such priming activates the care foundation, promoting moral concerns for the well-being of others. An alternative possibility, however, is that the increased giving in the dictator game reflects the activation of the fairness foundation. For instance, the most frequent behavior for religiously primed participants in Shariff and Norenzayan’s (2007) studies was to transfer exactly half of the available money (in accordance with a salient norm of fairness), whereas for control participants, the most frequent behavior was to transfer nothing. (This might be seen as compelling evidence that fairness concerns were paramount here. However, although the modal response was to transfer half of the money, some participants in the religious prime condition transferred *more* than half—strictly speaking, an *unfair* allocation.) A similar issue arises when considering the study of Pichon et al. (2007). These authors found that participants primed with positive religion words (e.g., “heaven”) collected more pamphlets advertising a charity organization than participants primed with neutral religion words (e.g., “parish”), positive words unrelated to religion (e.g., “liberty”), or neutral words unrelated to religion (e.g., “shirt”). One might conclude that religious priming (or, at least, *positive* religious priming) had activated compassion for the disadvantaged. But charitable behaviors or concerns could also be driven by an aversion to inequity (Fehr & Schmidt, 1999).

Notwithstanding these interpretive complexities, the results of religious priming studies, taken together, would seem to indicate that religious priming promotes adherence to moral norms. Nevertheless, the picture may be more complicated than this, as other studies have shown that religious priming also elicits a range of aggressive and prejudicial behaviors. For example, Bushman, Ridge, Das, Key, and Busath (2007) found that participants who read a description of violent retaliation commanded by God were subsequently more aggressive than participants who read the same description, but with the passage about God’s sanction omitted. Saroglou, Corneille, and Van Cappellen (2009) found that religiously primed participants encouraged by the experimenter to exact revenge on an individual who had allegedly criticized them were more vengeful than those given neutral primes. Van Pachterbeke, Freyer, and Saroglou (2011) found that religiously primed participants displayed support for impersonal societal norms even when upholding such norms would harm individuals (the effects reported by Saroglou et al. and Van Pachterbeke et al. were limited to participants scoring high on measures of submissiveness and authoritarianism, respectively). M. K. Johnson, Rowatt, and LaBouff (2010) found that subliminal priming of Christian concepts in ethnically diverse participant samples increased covert racial prejudice and negative affect toward African Americans (see also LaBouff, Rowatt, Johnson, & Finkle, 2012; Van Tongeren, Raad, McIntosh, & Pae, 2013). And Ginges et al. (2009) found that Jewish settlers were more likely to endorse as “extremely heroic” a suicide attack carried out against Muslims by an Israeli Jew when primed with synagogue attendance than when unprimed.

The fact that religious priming has been shown to elicit both “prosocial” and “nonprosocial” effects (Galen, 2012) is often viewed as something of a contradiction or inconsistency (e.g., Preston & Ritter, 2013; Saroglou, 2006). One might suppose that the effects of such priming on aggression and prejudice count against the hypothesis that intuitions about supernatural observers inhibit moral norm violations. But without knowing what participants perceive as the relevant norm, this is difficult to establish. It may be that the putative “nonprosocial” effects involve adherence to, rather than violation of, a perceived norm. For example, in the Bushman et al. (2007) study, God’s apparent sanctioning of violent retaliation may reasonably be perceived as establishing a religious norm that participants then adhere to by behaving aggressively (Preston & Ritter, 2013; see Blogowska & Saroglou, 2013).

There are other reasons to doubt that religious priming studies demonstrate that activating intuitions about punitive supernatural agents curbs moral infractions. For example, although Shariff & Norenzayan (2007) suggested that their primes had “aroused an imagined presence of supernatural watchers” (p. 807), Randolph-Seng and Nielsen (2008) argued that the use of primes that are semantically associated with moral behavior (e.g., “God”) may lead to moral behavior simply by virtue of that association. This “behavioral priming” interpretation of Shariff and Norenzayan’s results is consistent with their discovery, in their second study, that the effect on dictator game behavior of “secular” primes (civic, jury, court, police, and contract) was comparable with that of religious primes. Randolph-Seng and Nielsen ask why secular primes such as “civic” and “contract” should increase giving behavior if such behavior results from the activation of “supernatural watcher” concepts. The effect of the secular primes, they suggest, is more consistent with the behavioral priming explanation.

Similar considerations apply to a study by Mazar et al. (2008), who found that participants who wrote down the titles of 10 books they had read in high school cheated on a subsequent task if given the opportunity to do so, whereas participants who instead wrote down the Ten Commandments did not. In a second study, these authors found that a secular reminder of morality (a statement about the university’s honor code) had the same effect on cheating as the Ten Commandments prime. More recently, Ma-Kellams and Blascovich (2013) found that even primes of *science* (e.g., words such as “hypothesis,” “laboratory,” and “scientists”) promoted adherence to moral norms and morally normative behaviors (these researchers examined morality primarily in the harm–care and fairness domains).

McKay and Dennett (2009), however, have argued that the primes used in such cases do not enable clear adjudication between the surveillance and “behavioral priming” accounts. For example, both the “religious” and “secular” conditions in Shariff and Norenzayan’s (2007) second study included words associated not just with moral behavior but also with intelligent agents (“God” and “prophet” in the religious condition; “jury” and “police” in the secular condition). Gervais and Norenzayan (2012a) have recently shown that participants exposed to Shariff and Norenzayan’s religious primes showed a subsequent increase both in public self-awareness and socially desirable responding—two variables that are sensitive to the perception of being observed. This result seems an impressive substantiation of Shariff and Norenzayan’s supernatural watcher hypothesis. It remains to be demonstrated, however, that the perception that one is observed is what mediates the effect of the primes on behavior. It is possible that religious priming might activate both surveillance concerns *and* moral concepts, but that only the latter influence game behavior.^9^

Earlier we mentioned methods that potentially conflate distinct moral motivations (e.g., the care and fairness foundations). The contrast between care and fairness is perhaps starkest when considering retributive punishment (“an eye for an eye”) and forgiveness (“turn the other cheek”). Jesus preached the latter (e.g., Matthew 5:39; Luke 6:29), and in so doing arguably prioritized kindness and compassion over fairness and justice (the command to “turn the other cheek” is effectively an endorsement of “second-order” free riding; Panchanathan & Boyd, 2004).^10^ The dichotomy between forgiveness and punishment provides a potential empirical lever for teasing apart the effects of supernatural primes on kindness and fairness. What effect would such primes have on the altruistic punishment of unfair behavior (Fehr & Gächter, 2002)? If supernatural primes activate concerns for fairness, then primed participants should be more likely to punish violations of fairness norms. If, on the other hand, such primes stimulate kindness, then participants may be *less* likely to engage in such punishment.

A study by McKay, Efferson, Whitehouse, and Fehr (2011) bears on this question. Participants were primed subliminally with the concepts of *religion* and/or *punishment*, and the extent to which they subsequently punished unfair offers in a punishment game was measured. We found that religious primes strongly increased the costly punishment of unfair behaviors for a subset of our participants—those who had previously donated to a religious organization. This finding seems consistent with the notion that supernatural agency concepts promote fairness and its enforcement, although, as this study did not disambiguate agency and moral dimensions along the lines suggested earlier, it may be that the effect here was a result of behavioral priming of moral behavior (in this case, punishment of unfair behavior) rather than activation of supernatural agent concepts. Another problem is that different idiosyncratic conceptions of God (e.g., compassionate vs. punitive) may, when primed, result in very different behaviors. Earlier studies, for example, have found that whereas people who report having a close personal relationship with a loving god are less likely to support the death penalty (Unnever, Cullen, & Bartkowski, 2006), those who conceive of God as a powerful dispenser of justice are more likely to support the death penalty (Unnever, Cullen, & Applegate, 2005; see also Shariff & Norenzayan, 2011). When possible, therefore, priming studies should attempt to measure idiosyncratic conceptions of God (e.g., Laurin, Shariff, Henrich, & Kay, 2012).

Overall, we think that religious priming studies provide at least tentative evidence that activating intuitions about supernatural agents curbs moral norm violations. However, it is important to note that almost all of these studies were conducted in WEIRD societies (Henrich et al., 2010), typically using undergraduate student populations.^11^ The extent to which these effects generalize to other cultures is therefore unclear. But what of the intuitions themselves?

### The Cross-Cultural Prevalence of Supernatural Punishment Concepts

If intuitions about such supernatural punishers are properly *foundational*, they should be culturally and historically widespread. However, Baumard and Boyer (2013a) note that the gods of numerous classical traditions (e.g., Greek, Roman, Chinese, Hindu) “were generally construed as unencumbered with moral conscience and uninterested in human morality” (p. 272; see also Baumard & Boyer, in press; Schlieter, 2014). Further illustration of the cultural and historical variability in this respect comes from the Standard Cross Cultural Sample (SCCS),^12^ which sorts the variable “high gods” into four categories: (a) “absent or not reported,” (b) “present but not active in human affairs,” (c) “present and active in human affairs but not supportive of human morality,” and (d) “present, active, and specifically supportive of human morality” (Divale, 2000; see D. D. P. Johnson, 2005; Roes & Raymond, 2003). It seems clear that not all supernatural agents are explicitly represented as taking an interest in human morality: Insofar as the gods monitor human behavior, in many traditions this is primarily to oversee adherence to nonmoral strictures and the appropriate performance of costly rituals and sacrifices (Purzycki, 2011; Purzycki & Sosis, 2011).

Although these considerations may seem to refute any suggestion that moralizing, punitive supernatural agents are historically and cross-culturally universal, recent work suggests that even when gods are not explicitly represented as caring about human morality, there is nevertheless a moral undercurrent beneath the surface of such explicit, reflective representations (Purzycki, 2013). For example, ethnic Tyvans (from the central Asian Republic of Tuva) rate spirit masters’ knowledge and concern about moral information (e.g., theft) higher than nonmoral information (Purzycki, 2013), despite explicitly denying that spirit masters care about interpersonal moral behavior (Purzycki, 2010).

In any case, as Graham et al. (2013) argue, foundationhood does not require that the foundation in question be shown to underlie relevant cultural representations in all human cultures. Cultural influences may restrict the expression of innate cognitive tendencies, just as they can restrict the expression of innate physical propensities (e.g., foot binding in Imperial China restricted the growth of the feet; Ko, 2002). However, Graham and colleagues also note that not all cultures are equally informative when it comes to establishing foundationhood. In particular, the most informative societies are those most closely resembling relevant ancestral lifestyles (Tooby & Cosmides, 1990, 1992; see Marlowe, 2005). And it is in these small-scale hunter–gatherer societies that explicit doctrines about moralizing, punitive supernatural agents are conspicuously absent (Baumard & Boyer, 2013a; Boehm, 2008; Boyer, 2001). For example, the Hadza of northern Tanzania and the !Kung of the Kalahari Desert are contemporary hunter–gatherer societies with gods who take little interest in human wrongdoing (Norenzayan, 2013).

In our judgment, therefore, it is unlikely that our evolved cognitive systems produce stable intuitions about omnipresent supernatural punishers. What we think more plausible is that we have a genetically endowed sensitivity to situational cues that our behavior is being observed. Experiments demonstrate that people—even young children—are “strategically prosocial,” behaving more generously and cooperatively when they know others can observe their behavior (e.g., Gächter & Fehr, 1999; Leimgruber, Shaw, Santos, & Olson, 2012; Wedekind & Milinski, 2000). A burgeoning literature indicates that even very subtle cues of surveillance influence adherence to prevailing moral norms. For example, Haley and Fessler (2005) found that the presence of stylized eye-like images on the computer background had a substantial influence on the number of participants who, under conditions of strict anonymity, allocated money to another individual in a computerized dictator game (nearly 80% of participants in the “eyespots” conditions transferred money, compared with just over 50% in conditions without eyespots). Rigdon, Ishii, Watabe, and Kitayama (2009) replicated this experimental result using three dots in a schematic face configuration, compared with a condition in which this configuration was reversed vertically (see also Baillon, Selim, & van Dolder, 2013; Burnham & Hare, 2007; Oda, Niwa, Honma, & Hiraishi, 2011; cf. Fehr & Schneider, 2010). In contrast to these studies, Raihani and Bshary (2012) found that dictators donated *less* money in the presence of eye images. However, these authors only analyzed mean donations, and not the probability of donating something (however small). Nettle et al. (2013) argue that the reliable effect of surveillance cues in the dictator game is to increase the probability that dictators will donate something, rather than to increase mean donations. A reanalysis by these authors of Raihani and Bshary’s (2012) data confirmed the former effect.

Bateson, Nettle, and colleagues have found similar effects using an image of a pair of eyes on a notice in naturalistic settings. Bateson, Nettle, and Roberts (2006) found that, compared with images of flowers, eye images substantially increased the level of contributions to an honesty box in a psychology department tea room; and Ernest-Jones, Nettle, and Bateson (2011) found that similar images halved the odds of littering in a university cafeteria. Bourrat, Baumard, and McKay (2011) found that such images led to greater *condemnation* of moral infractions. Relatedly, Cavrak and Kleider-Offutt (2014) recently found that participants exposed to religious images associated with a prominent supernatural agent (e.g., a crucifix, a crown of thorns, a Jesus Fish or Ichthys) rated morally ambiguous actions as less morally appropriate than did participants exposed to control images. Finally, there is evidence that experimental cues of *anonymity* rather than of surveillance (e.g., dimmed lighting, the wearing of sunglasses) led to *more* moral infractions (Zhong, Bohns, & Gino, 2010). Tane and Takezawa (2011) found that Haley and Fessler’s (2005) stylized eye-like images had no effect on dictator game allocations when the stimuli were presented in a dark room.

The upshot of all this work is that evolved agency-detection mechanisms may serve to deliver intuitions about observing agents *and* to regulate our behavior in the presence of those agents. We doubt, however, that such mechanisms deliver intuitions about moralizing, punitive supernatural agents—instead, we think that the relevant intuitions are more basic (just concerning the presence of agency per se). Triggered in the absence of any visible intentional agent, however, such intuitions may be *reflectively elaborated* into conclusions about supernatural watchers (Baumard & Boyer, 2013b). And drawing on intuitions about fairness and the psychological characteristics of intentional agents (ToM), such supernatural watcher concepts may morph into more complex, compelling, and culturally transmissible notions of moralizing gods—notions which, when made salient or activated (as in priming studies), serve to promote adherence to the perceived norms of those gods.

Here we see the essential arbitrariness of the “religion” and “morality” categories, for there may be considerable overlap between “religious” and “moral” features at the levels of both cognitive predispositions and cultural representations. After all, it is clear that cultural representations of morally concerned, punitive supernatural agents—“moralizing gods” (Roes & Raymond, 2003)—are both religious *and* moral. Moreover, the notion that cultural notions of such gods are undergirded by cognitive intuitions about agency, ToM, and fairness (or “proportionality”; Baumard & Boyer, 2013a) is not just plausible but also compelling.

What this highlights is that we can often make no principled distinction between religion and morality at the level of culture *or* cognition. Our aim here has been to pinpoint some of the major features in the religious and moral constellations. When we play the astrologer’s game, in considering the biological and cultural interplay between certain—essentially arbitrary—sets of these features, we do so in order to engage and accommodate our academic colleagues. Ultimately, however, we see evolved cognitive systems for care, fairness, loyalty, respect, and purity as “religious foundations” no less than as “moral foundations.” A thoroughgoing science of “religion” and “morality” may ultimately dispense with these terms, exhaustively mapping the relations between evolved cognitive systems and cultural representations without recourse to vague overarching labels.

## The Religion–Morality Relationship in Cultural Evolution

Recall the analogy drawn earlier between the properties of (a) hands and gloves, and (b) evolved cognitive systems and explicit cultural representations. Whereas hands are biologically evolved features of human anatomy, gloves are culturally evolved artifacts that must follow the contours of the hand at least to some extent in order to be wearable. In this section, we ask whether, in a similar fashion, culturally evolved belief systems must follow the contours of our evolved cognitive systems. Moreover, from the perspective of our concern with the religion–morality relationship, do cultural systems create durable connections between the moral and religious foundations depicted in Figure 2? Do religious cultural representations influence the prevalence of moral cultural representations and/or do they constrain the activation of moral intuitions? In posing these particular questions, we do not mean to suggest that the direction of causality must always run from religion to morality. It could be that “moral” cultural representations amplify or constrain the activation of “religious” intuitions. For example, a sign in a public restroom designed to encourage hand washing by reminding people of a behavioral norm (“Is the person next to you washing with soap?”) may trigger intuitions about observing agents (Judah et al., 2009).

In considering these questions, one might seek to supplement the examples in Figure 2 with further examples plucked from the ethnographic record. Although time-consuming, such an exercise would undoubtedly be instructive in many ways. It would indicate, for example, whether—and how—cultural systems from diverse regions of the world are capable of connecting moral and religious foundations in a variety of ways. It would not, however, address the deeper question of why they do so. To examine the “how” question, we provide a case study based on long-term immersion in a particular cultural system. To examine the “why” question, we consider two competing perspectives: a cultural adaptationist account and a cultural epidemiological one.

### The Pomio Kivung: A Case Study in Culturally Evolved Connections Between Religious and Moral Foundations

To illustrate some of the ways in which cultural systems may serve to connect the fractionated elements of religion and morality (the “how” question), we consider a cargo cult in East New Britain Province, Papua New Guinea, known as the Pomio Kivung (Whitehouse, 1995). In *Tok Pisin* (the lingua franca of Papua New Guinea), the word *Kivung* means “a meeting” or “to meet,” but for several ethnic groups in New Britain, it also designates a popular religious movement. Established in the early 1960s and spreading to encompass scores of villages in some of the more remote regions of the island, the movement has a centralized leadership, based at a large coastal settlement, from which regular patrols to outlying villages are sent, bringing news, collecting taxes, and policing the orthodoxy. Each Kivung village has a team of designated orators, trained at the movement’s headquarters, charged with the responsibility of preaching a standard body of doctrines and overseeing a wide range of authorized rituals. The mainstream Kivung exhibits all the fractionated elements of our intuitive religious repertoire: hyperactive agency detection, ToM, teleofunctional reasoning, the ritual stance, and group psychology. And it connects each of these elements to our five moral foundations (care, fairness, loyalty, respect, and purity).

At the heart of Kivung teachings is the idea that the ancestors of followers will someday soon return from the dead, bringing with them all the wonders of Western technology. Until that day, however, the ancestors exist only as bodiless agents, discernible by the sounds they make and the traces they leave behind. The ancestors are believed to mill around with the living as they go about their daily activities, invisibly observing people’s comings and goings, and taking a particular interest in the moral implications of their behavior. Failures to observe the laws of the Kivung are said to delay the miracle of returning ancestors. Only when a certain moral threshold has been achieved will the living and the dead be reunited. This dogma connects with all our moral foundations because the Kivung laws, adapted from the Ten Commandments as taught by Catholic missionaries in the region, forbid such a broad range of transgressions as violence and slander (harming), cheating and stealing (fairness), criticizing the Kivung (loyalty), disobedience (respect), and cooking during menses (purity).

Kivung ideas about ancestors not only link up our moral foundations but also weave intricate connections through discourse and ritual between each of our religious foundations. For example, among the many rituals observed by Kivung followers is the daily laying out of food offerings to the ancestors. Great attention is paid to the noises of ancestors entering the temple (e.g., the creaking of the door), tampering with the food (e.g., the clattering of dishes), and the visible signs of eating (e.g., morsels of food apparently removed by invisible hands). These ideas obviously prime agency detection—moreover, there is a specialist (whose official role translates roughly as “witness”) charged with responsibility for observing vigils in the temples and listening for signs of invisible ancestral presence. Insofar as ancestors are said to be able to see into people’s hearts and minds, Kivung dogma presents formidable ToM challenges and a suite of rituals dedicated to assuaging feelings of guilt and shame, as well as the pursuit of forgiveness and absolution. A common way of paying for one’s sins to is place money into a special receptacle or (because not all Kivung followers have access to money) to place one’s hand over the receptacle to display the intention to give. This simple ritual requires intense concentration, as it is said that if the ancestors detect insincerity (telepathically), they will withhold their forgiveness. Teleofunctional reasoning meanwhile is a pervasive feature of Kivung origin myths and various rituals associated with the sacred gardens (one of which memorializes a Melanesian Eden). And lastly, the Kivung activates group psychology by creating familial ties based on shared ritual experiences and coalitional bonds via us–them thinking in relation to external detractors and critics.

Although the Kivung connects up all our moral and religious foundations through a highly elaborated system of doctrines and practices, many of which borrow liberally from missionary teachings, we cannot assume that the same would be true of all cultural systems typically classified as “religious.” This is a matter for anthropologists to establish on a case-by-case basis. In the end, however, it constitutes a question about *how*, rather than *why*, cultural systems create connections between moral and religious foundations. To address the why, we need to consider issues of function and ultimate causation.

### Adaptationist and By-Product Accounts

Two contrasting positions on the why of the morality–religion relationship in cultural evolution have achieved some prominence in recent years. One takes the form of adaptationist arguments concerning the emergence and spread of routinized rituals and moralizing gods. The other argues that all cultural traditions, however they trace (or fail to trace) the connections between moral and religious foundations, are by-products of cognitive predispositions and biases, rather than cultural adaptations that enhance the fitness of individuals or groups. We briefly review these alternative positions and consider what evidence would be required to adjudicate satisfactorily between the two.

Scholars in the cognitive science of religion tend to agree that many globally and historically recurrent features of religious thinking and behavior are by-products of cognitive machinery that evolved for reasons that have nothing to do with religion (e.g., Atran, 2002; Atran & Norenzayan, 2004; J. L. Barrett, 2004; Bloom, 2009; Boyer, 2001). For example, HADDs are thought to have evolved to help support the detection of predators and prey. If they also undergirded intuitions about the presence of bodiless agents, then this was originally a side effect (by-product) of their main function (J. L. Barrett, 2000, 2004, 2012). To express this in terms of our body–clothing analogy, if HADDs were equivalent to the evolved anatomy of the hand, then the accumulated cultural knowledge of expert trackers and hunters would be equivalent to the protective functions of gloves, essential for survival in very cold climates. But gloves can also have decorative frills, like bobbles and tassels, which have no particular survival value. Cultural representations concerning bodiless agents would be decorative frills of this kind. As such, these kinds of functionally superfluous additions need not follow the contour of the hand at all—and might derive their popular appeal precisely from the fact that they do not. Thus, one of the dominant explanations for the cultural recurrence of supernatural agent concepts is that they violate intuitive expectations in ways that are especially attention grabbing and memorable—like glittering jewels adorning the gauntlet of an emperor (Boyer, 2001; Pyysiäinen, 2001). Conceivably, the cultural success of certain Christian ideals (e.g., “turning the other cheek”) may owe, in part, to the fact that they violate intuitions about proportionality (“an eye for an eye”).

What distinguishes the adaptationist perspective on religion, however, is the view that at least some of these religious by-products became useful for the survival of individuals and groups in the course of cultural evolution. Most commonly, this argument has been applied to the growth of large-scale societies. Humans evolved to live in face-to-face bands of hunter–gatherers rather than in vast empires or nations. Small group psychology, it has been argued, would have been insufficient to handle many of the challenges of large group living. Religion provided cultural adaptations to support the transition from foraging to farming, from local community to state formation. One line of adaptationist thinking has focused on the role of ritual frequency in this transition (Whitehouse, 2012). As collective rituals came to be performed more regularly, beliefs and practices that defined the group could be standardized across larger populations, a tendency that was reinforced by the invention of literacy (Mullins, Whitehouse, & Atkinson, 2013). As common identity markers came to unite ever larger coalitions, local communities bound together by small group psychology tended to be engulfed and absorbed or wiped out altogether (Turchin, Whitehouse, Francois, Slingerland, & Collard, 2012). Another line of adaptationist thinking has focused on the role of rituals as costly signals and “credibility enhancing displays.” Still another has focused on the role of moralizing gods in the evolution of social complexity. We consider each of these approaches in turn.

#### Routinization

One of the major challenges in understanding how and why religion changes as societies become larger and more complex relates to the changing structure and function of ritual. As conditions permitted an escalation of the scale and complexity of human societies, cultural evolutionary processes may have further tuned the elements of ritual, promoting social cohesion. With the evolution of social complexity, religious rituals become more routinized, dysphoric rituals become less widespread, doctrine and narrative becomes more standardized, beliefs become more universalistic, religion becomes more hierarchical, offices more professionalized, sacred texts help to codify and legitimate emergent orthodoxies, and religious guilds increasingly monopolize resources (Whitehouse, 2000, 2004). Some of these patterns have recently been documented quantitatively using large samples of religious traditions from the ethnographic record. For instance, Atkinson and Whitehouse (2011) have shown that as societies become larger and more hierarchical, rituals are more frequently performed (Atkinson & Whitehouse, 2011), and low-frequency dysphoric rituals typical of small, cohesive social groups, such as warring tribes (Whitehouse, 1996), come to be confined to specialized niches (e.g., hazing and initiation in military organizations). Small, tightly bonded groups with dysphoric rituals may be generally deleterious to cooperation in larger societies (creating opposing coalitions), and thus “selected out” of the cultural repertoire, at least for the population at large, and relegated to confined organizations (e.g., militaries). Instead, the much more frequent rituals typical of regional and world religions sustain forms of group identification better suited to the kinds of collective action problems presented by interactions among strangers or socially more distant individuals (Whitehouse, 2004). As rituals become more routinized, however, they also become less stimulating emotionally, and perhaps even more tedious (Whitehouse, 2000). New rituals then evolved in some traditions to convey propositional information about supernatural beliefs through a combination of repetition and costly displays (such as animal sacrifices or monetary donations) that culturally transmit commitment to certain beliefs (Atran & Henrich, 2010; Henrich, 2009). As some societies became ever larger and more complex, even the processes described here may not have been sufficient to sustain cooperation and a host of new cultural adaptations—most notably, forms of external information storage and secular institutions of governance—became increasingly important (Mullins et al., 2013; Norenzayan, 2013).

#### Costly signaling and “credibility-enhancing displays.”

“Costly signaling” theorists have argued that rituals serve as a hard-to-fake index of commitment to the group (Irons, 2001). Although originally used by biologists to denote the display of costly signals of fine health, such as the peacock’s tail or the leaping of springbok (Grafen, 1990; Zahavi & Zahavi, 1997), applications of signaling theory to ritual behavior in humans adopt a broader conception of “costliness”—in terms of time, labor, money, goods, and health (Bulbulia, 2008; for a critique, see Murray & Moore, 2009). To avoid confusion with the narrower meaning of costly signaling in biology, some social scientists prefer to talk of “commitment signaling” or “honest signaling” (Bliege Bird & Smith, 2005; Sosis & Alcorta, 2003). With the emergence of agriculture and larger, more complex social formations, strangers (or relative strangers) needed to be able to assess their respective reputational statuses when biographical information was not readily available. It has been argued that rituals provided a signal of good character (trustworthiness and willingness to cooperate) in the absence of specific information about other people’s personal histories (Bulbulia et al., 2013).

The signaling theory of religion and ritual has been recently extended by the theory of credibility enhancing displays (CREDS; Henrich, 2009). By engaging in costly behaviors, rather than merely advocating such behavior in others (i.e., by “walking the walk” as well as “talking the talk”), role models secure the trust and devotion of followers. This is thought to facilitate the spread of moral norms across large populations and safeguard their transmission across the generations. CREDS theory seeks to explain not only the wide distribution of moral norms in the so-called ethical religions but also the prevalence of moral exemplars in such traditions (e.g., gurus, prophets, priests, and messiahs) and the willingness of rulers to be bound by the divine edicts.

#### The cultural evolution of “moralizing gods.”

One of the most vigorous debates in the recent literature on religion and morality has concerned the cultural prevalence of moralizing gods—powerful supernatural agents who monitor behavior and punish moral infractions. Ara Norenzayan and colleagues (e.g., Norenzayan, 2013, 2014; Norenzayan & Shariff, 2008; Norenzayan, Shariff, & Gervais, 2009; Shariff, Norenzayan, & Henrich, 2009) have argued that the cultural innovation of notions of such gods over the last 12 millennia has been an important factor in the human transition from small-scale, kin-based groups to large-scale societies.

In small-scale and traditional societies in which everybody knows everyone else and most social behavior is easily observed and reported, transgressions are easily detected. Modern technologies of surveillance, such as police cameras, identity cards, and computer records, allow increasingly extensive monitoring of thieves, cheats, defectors, and free riders by designated authorities. But for several thousand years, during which the so-called “ethical religions” evolved, much of the world’s population has lived in relatively complex societies in which interactions with strangers were common and parasitic free riders could evade punishment by wearing the cloak of anonymity. According to Norenzayan and colleagues, the postulation of moralizing gods provided an “eye in the sky” (Gervais & Norenzayan, 2012a), curtailing the deleterious effects of free riders and cheats, and allowing groups with such gods to survive and prosper, in turn enhancing the spread of the relevant god notions. Norenzayan et al.’s theory is thus (cultural) adaptationist in nature, as it claims that the cultural success of moralizing god concepts is partly a result of the adaptive effects of such concepts on human groups.

In contrast, Baumard and Boyer (2013a) argue incisively that the cultural prevalence of moralizing god representations does not result from the fact that such representations promote socially cohesive behaviors among human groups. Instead, these representations are successful because they have features (e.g., resonance with stable intuitions about proportionality and with elaborated intuitions about invisible agency) that render them especially attention-grabbing, memorable, and transmissible. In short, moralizing gods are cultural variants with effects that enhance their own success (and so are adaptive in that sense; Dennett, 1995), but these effects do not include changes in the biological or cultural fitness of their human vectors.

How are we to evaluate these opposing views? One feature of Norenzayan et al.’s position is that it seems to entail that supernatural agent representations should promote moral behaviors in the relevant cultures. As we have seen, a wealth of evidence from priming studies indicates that the activation of supernatural concepts can promote adherence to moral norms. On the other hand, other priming studies have revealed “nonprosocial” effects of religious primes (Galen, 2012). Do the latter studies undermine the hypothesis of Norenzayan and colleagues?

In our view, the tension between the “prosocial” and “nonprosocial” effects of religious primes may be a consequence of a sanitized conception of “prosociality.” The contention of Norenzayan and colleagues is that the cultural success of “moralizing gods” owes to the fact that members of groups with beliefs in such gods engage in behaviors that allow those groups to become larger and larger—that favor their “stability, survival, and expansion, at the expense of less successful rivals” (Norenzayan, 2013, p. 30). Such behaviors are literally “prosocial,” but we should not expect them to be “prosocial” in the sanitized social psychological sense. On the contrary, they may be aggressive, murderous, and even genocidal. Activating the notion of moralizing supernatural agents should encourage behaviors that advance the interests of the ingroup, whether these behaviors are “nice” or “nasty.” When priming with god concepts promotes altruism, we should expect this altruism to be parochial (confined to the ingroup) rather than indiscriminate (Hartung, 1995), and we should not be surprised if behaviors are undertaken to damage relevant out-groups (Blogowska et al., 2013; De Dreu et al., 2010). In short, attempts to substantiate Norenzayan’s theory with evidence of “religious prosociality” (the sanitized kind) may be misguided.

The pattern of “prosocial” and “nonprosocial” findings that has emerged from priming studies, to date, is quite consistent with Norenzayan’s theory. It is less clear that these findings are consistent with Baumard and Boyer (2013a). The latter authors claim that the success of moralizing god concepts is entirely a result of the resonance of these concepts with the output of intuitive systems, so their theory does not require that these concepts have any effects whatsoever on behavior. Any such effects are incidental and superfluous from their perspective.

In making their case, Baumard and Boyer argue that the gods of many prominent historical large-scale societies were “strikingly nonmoral”:

To simplify somewhat, the Romans, with their nonmoralizing gods, built one of history’s most successful predatory empires. They then converted to Christianity, a moralizing religion, and were promptly crushed by barbarians with tribal, nonmoralizing gods. (Baumard & Boyer, 2013a, p. 276)

Baumard and Boyer thus argue that moralizing religions were not the “magic bullet” enabling the formation of large-scale societies. A potential limitation of their formulation, however, is that they appear to identify gods as “nonmoralizing” if those gods are not explicitly represented as caring about human morality. As they acknowledge, however, the gods of antiquity were represented as monitoring the appropriate performance of rituals. To the extent that rituals represent or promote moral behaviors (see earlier), therefore, gods that care about rituals care about morality, directly or indirectly. We note in this connection that common components of ritual performance may facilitate parochially altruistic behaviors, including aggression (e.g., Wiltermuth, 2012, has recently shown that participants who acted in synchrony with a confederate were more likely to comply with the confederate’s request to administer a blast of noise to other participants than were control participants). In sum, in our view, a full evaluation of cultural evolutionary hypotheses about the connection between religion and morality requires reorientation on at least two fronts: what is important is that notions of the relevant gods promote socially cohesive behaviors, not that the behaviors are “nice,” and not that the gods are explicitly represented as valuing social cohesion.

## Conclusion

The relationship between religion and morality is a deep and emotive topic. The confident pronouncements of public commentators belie the bewildering theoretical and methodological complexity of the issues. In the scholarly sphere, progress is frequently impeded by a series of prevailing conceptual limitations and lacunae. Many contemporary investigations employ parochial conceptions of “religion” and “morality,” fail to decompose these categories into theoretically grounded elements, and/or neglect to consider the complex interplay between cognition and culture. The tendency to adopt a sanitized conception of prosocial behavior has hampered efforts to test theories of the extraordinary cultural dominance of “moralizing god” concepts—as we have seen, behaviors that allow religious groups to survive and expand may be anything but “nice.”

We have set out an encompassing evolutionary framework within which to situate and evaluate relevant evidence. Our view is that cultural representations—concepts, dogmas, artefacts, and practices both prescribed and proscribed—are triggered, shaped, and constrained by a variety of foundational cognitive systems. We have sought to identify the most currently plausible conjectures about biologically evolved connections between these systems, and have reviewed and evaluated the most prominent published debates in the cultural evolutionary domain. Ultimately, we see and foresee no pithily characterizable relationship between religion and morality. First, to the extent that the terms “religion” and “morality” are largely arbitrary and do not refer to coherent natural structures (as we have suggested), efforts to establish connections between religion and morality, conceived as monolithic entities, are destined to be facile or circular (or both). Second, under the pluralistic approach we advocate, which fractionates both religion and morality and distinguishes cognition from culture, the relationship between religion and morality expands into a matrix of separate relationships between fractionated elements. Thus some aspects of “religion” may promote some aspects of “morality,” just as others serve to suppress or obstruct the same, or different, aspects. In short, in discussing whether religion is a force for good, we must be very clear what we mean by “religion” and what we mean by “good.”

Although we eschew a simplistic story, we live in a very exciting time for psychological research on this topic. A key avenue for future work is to establish which biologically endowed cognitive structures and preferences are truly foundational where “religion” and “morality” are concerned. The aim should be to settle upon a parsimonious set of culturally and historically widespread cognitive predispositions that exhibit developmental and comparative evidence of innate preparedness, and that jointly account for the great bulk of culturally distributed items falling under the umbrella of religion and morality. In the meantime, taking into consideration data from non-WEIRD populations (Henrich et al., 2010), empirical work seeking to clarify relationships between religious and moral concepts and behaviors should capitalize on this fractionating approach by expanding the domain of relevant variables (for recent studies that have delineated a range of moral outcomes in accordance with MFT, see Cavrak & Kleider-Offutt, 2014; Gervais, 2014a). In particular, researchers should seek to characterize the range of “prosocial” outcomes (including outgroup aggression and hostility) more comprehensively, and when possible, should distinguish between parochial and more generalized variants of altruistic behaviors (e.g., Reddish, Bulbulia, & Fischer, 2013; Smith, Aquino, Koleva, & Graham, 2014). Research on “religion” and “morality” proceeds apace, but to capitalize on the gains that have been made, we must adopt higher standards of conceptual precision—a hallmark of maturation in any field of science.

## Figures and Tables

**Figure 1 fig1:**
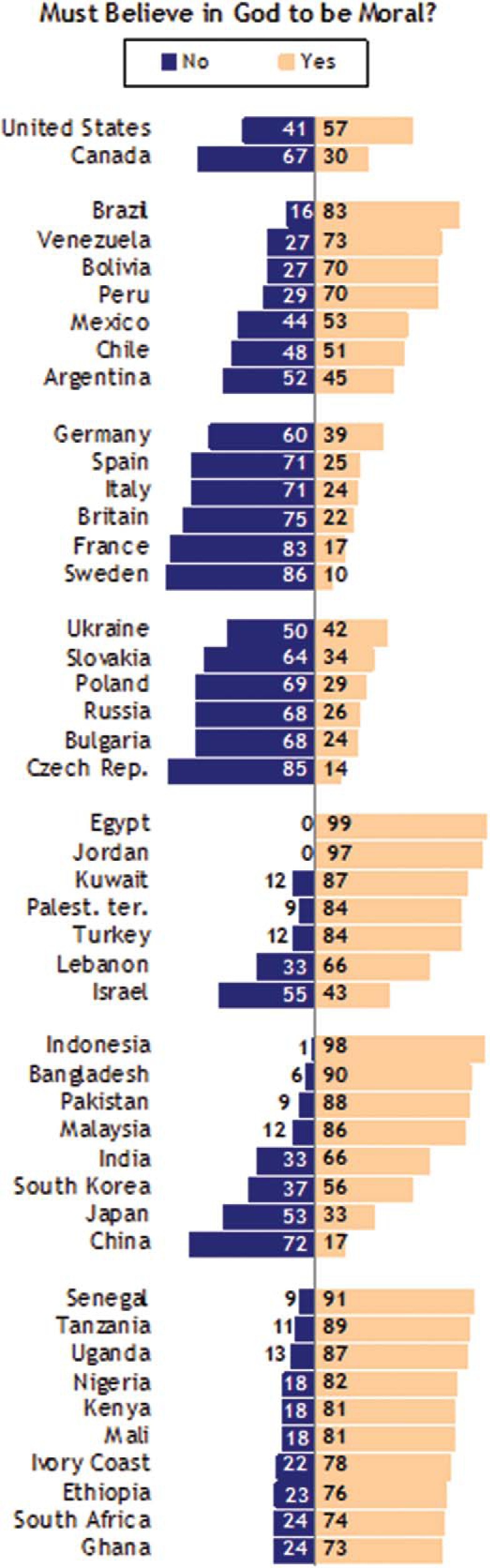
Views of religion and morality (Pew Research Center, 2007; reprinted with permission). See the online article for the color version of this figure.

## Footnotes

1 Here we conflate two different senses in which morality may require God. On the one hand, morality may require God in the sense that the very notion of morality is incoherent without God (i.e., without God, there is no basis for ethics). This is what Socrates had in mind (and disputed). On the other hand, morality may require God in the sense that (belief in) God is needed to enforce moral behavior. This is what Dostoevsky meant. Partly for rhetorical effect, here we have presented the Socrates view and the Dostoevsky view as opposing, but strictly speaking, they could both be valid—for example, it could be that the notion of morality is coherent without God (Socrates), but that the threat of God’s punishment is required for anybody to actually act morally (Dostoevsky).

2 At the same time, atheists and believers alike view good deeds as less moral if they are performed for religious reasons (Gervais, 2014b).

3 A. B. Cohen and colleagues (e.g., A. B. Cohen, 2003; A. B. Cohen & Rankin, 2004; A. B. Cohen & Rozin, 2001) have investigated how different religious traditions vary with respect to the moral status accorded to thoughts. Some religions (e.g., Protestantism) view thoughts as morally equivalent to actions, whereas others (e.g., Judaism) do not.

4 Although Tinbergen apparently did not mention Aristotle in his work (Hladký & Havlíček, 2013), a number of authors have commented on the parallels between Tinbergen’s Four Whys and Aristotle’s teaching of Four Causes (e.g., L. Barrett, Blumstein, Clutton-Brock, & Kappeler, 2013). The point that scientific research on religion should consider all four whys has been eloquently made by Hinde (2005) and informs his writings on religion more generally (e.g., Hinde, 1999).

5 An advantage of our hand–glove analogy over the foundation–building analogy is that the morphologies of hands *suggest* gloves more than the morphologies of foundations suggest resulting architectural forms.

6 All undergraduate psychology students learn that correlation does not imply causation. This lesson is particularly important when considering evidence germane to the religion–morality debate. To illustrate, Brañas-Garza, Espín, and Neuman (2014) used economic games such as the dictator game (see Footnote 7) to explore the relationship between individual religious variables and morally relevant social behaviors (e.g., altruism, fairness) in a large Spanish sample. Although they found a positive relationship between intensity of religiosity and altruism in the dictator game, they acknowledged that the causality of this relationship could have run from altruism to religiosity, or that unobserved third variables may have influenced both altruism and religiosity.

7 The dictator game is an anonymous, two-player “game” in which the first player must decide how much of a monetary endowment to distribute to the second player. The second player has a completely passive role (which is why the dictator game is not, strictly speaking, a game) and must accept whatever the first player transfers. In a public goods game, players privately choose how much of an endowment to donate to a public pot. Total donations are subsequently multiplied by some factor (greater than 1 but less than the number of players) and this “public good” payoff is then distributed evenly among all players (a common-pool resource game is similar, but players choose how much *to withdraw* from a collective pot; if total withdrawals exceed the amount in the pot, no player receives anything). A prisoner’s dilemma game is essentially a simplified public goods game played with two players.

8 Although some authors have suggested that moral conduct of any kind may be impossible without self-control (e.g., according to Baumeister and Exline [1999, p. 1175], “it is fair to consider self-control the master virtue”), the relationship between self-control and morality is complex. For example, punishment of unfairness has been associated both with self-control (e.g., Knoch, Pascual-Leone, Meyer, Treyer, & Fehr, 2006; Lakshminarayanan & Santos, 2009) and with its opposite—impulsivity (e.g., Crockett, Clark, Lieberman, Tabibnia, & Robbins, 2010; Pillutla & Murningham, 1996; see Espín, Brañas-Garza, Herrmann, & Gamella, 2012). At present, there is no official moral foundation of self-control.

9 Ritter and Preston (2013) conducted a sophisticated recent investigation of lay understandings of religious prime words, finding evidence for the cognitive representation of three relatively distinct classes of religious concept: agent concepts (e.g., god, angel), spiritual–abstract concepts (e.g., faith, belief), and institutional–concrete concepts (e.g., shrine, scripture).

10 In contrast, Dante’s persona in the *Divine Comedy* appears to relish the suffering of the sinners who are experiencing divine justice in hell:
And I: “Master, I would be most eager To see him pushed deep down into this soup Before we leave the lake” Soon I watched him get so torn to pieces By the muddy crew, I still give praise And thanks to God for it. (*Inferno*, VIII: 52–54; 58–60; Alighieri, 1472/2002)

11 Two exceptions are Hadnes and Schumacher (2012) and Aveyard (2014). Hadnes and Schumacher found that priming West African villagers with traditional beliefs substantially increased trustworthy behavior in an economic trust game. Aveyard tested a sample of Middle Eastern Muslim undergraduates and found that whereas a laboratory priming manipulation had no effect on their cheating rates, participants exposed to a naturalistic religious prime—the Islamic call to prayer—cheated substantially less.

12 The SCCS is a database of 186 well-documented human societies, spanning contemporary hunter–gatherers, early historic states, and contemporary industrial societies. The sample was devised by Murdock and White (1969) and selected such that the included cultures capture the world’s regions and diversity, yet have relatively weak phylogenetic and cultural diffusion relationships to one another (thus avoiding “Galton’s problem,” whereby cross-cultural comparisons can generate spurious correlations if common attributes have been transmitted between societies or are descended from a common ancestor; D. D. P. Johnson, 2005). The database contains quantitative variables describing numerous characteristics of the societies in the sample. The “high gods” variable is defined by Murdock (1967, p. 52) as “a spiritual being who is believed to have created all reality and/or to be its ultimate governor, even though his sole act was to create other spirits who, in turn, created or control the natural world.”
